# Redox Chemistry and Photophysics of the **[V(dgpy)**_**2**_ **]** ^**3+/2+**^ Redox Pair

**DOI:** 10.1021/acs.inorgchem.6c00836

**Published:** 2026-05-23

**Authors:** Alexandra König, Marietta Goetz, Robert Naumann, Christoph Förster, Jan Klett, Maximilian E. Huber, Philipp Weber, Christoph Riehn, Jennifer Meyer, Katja Heinze

**Affiliations:** † Department of Chemistry, 9182 Johannes Gutenberg University Mainz, Duesbergweg 10-14, 55128 Mainz, Germany; ‡ Fachbereich Chemie und Forschungszentrum OPTIMAS, 26562 Rheinland-Pfälzische Technische Universität Kaiserslautern-Landau (RPTU), Erwin-Schrödinger Str. 52, 67663 Kaiserslautern, Germany

## Abstract

Transition-metal complexes with earth-abundant metal centers hold significant potential for light-induced processes. Pseudo-octahedral vanadium­(III) and vanadium­(II) complexes with d^2^ and d^3^ electron configurations are promising candidates, yet their redox and excited-state properties are only insufficiently understood. Here, we combine quantum chemistry and experimental data (structure, cyclic voltammetry, spectroelectrochemistry, electron-transfer dissociation, collision-induced dissociation, ultraviolet photodissociation mass spectrometry, and ultrafast transient absorption spectroscopy) to elucidate redox and photostability as well as excited-state lifetimes of the vanadium­(III/II) redox couple *cisfac* **-[V­(dgpy)**_**2**_ **]** ^**3+/2+**^ (dgpy = 2,6-diguanidylpyridine). Ground-state splitting, mixing of spin-flip excited states with metal-to-ligand charge transfer states, and multiphonon relaxation are identified as the key challenges for excited-state lifetimes in these d^2^/d^3^ systems.

## Graphical abstract

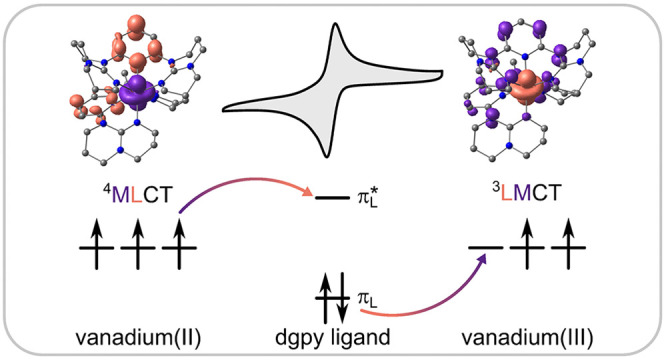

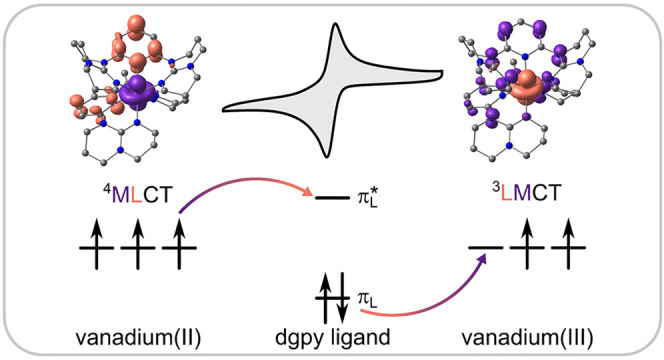

## Introduction

Exploration and exploitation of pseudo-octahedral polypyridyl vanadium­(II) and vanadium­(III) complexes^1–12^ for photophysical^13–20^ and redox applications has achieved less attention than the investigation and applications of polypyridyl chromium­(III) complexes.^14,17,18,21–36^ However, the first NIR-II luminescent d^2^-vanadium­(III) and d^3^-vanadium­(II) complexes [V­(ddpd)_2_]^3+^,^4^ V­(C_6_F_5_)_3_tren­(CN^*t*^Bu),^5^ VCl_3_(ddpd),^7^ and [V­(tpe)_2_]^2+^ ^12^ (Chart 1) have been recently reported, yet different ligand types are employed for the different oxidation states (ddpd = *N*,*N*′-dimethyl-*N*,*N*′-dipyridine-2-yl-pyridine-2,6-diamine,^37^ tpe = 1,1,1-tris­(pyrid-2-yl)­ethane^38^). In any case, the foremost prerequisite for spin-flip (SF) emission from octahedral d^2^ and d^3^ metal complexes is a sufficiently large ligand field splitting, so that interconfigurational ligand field states are well above the (intraconfigurational) SF states.^16^ Hence, strong-field ligands are required.

**Chart 1:**
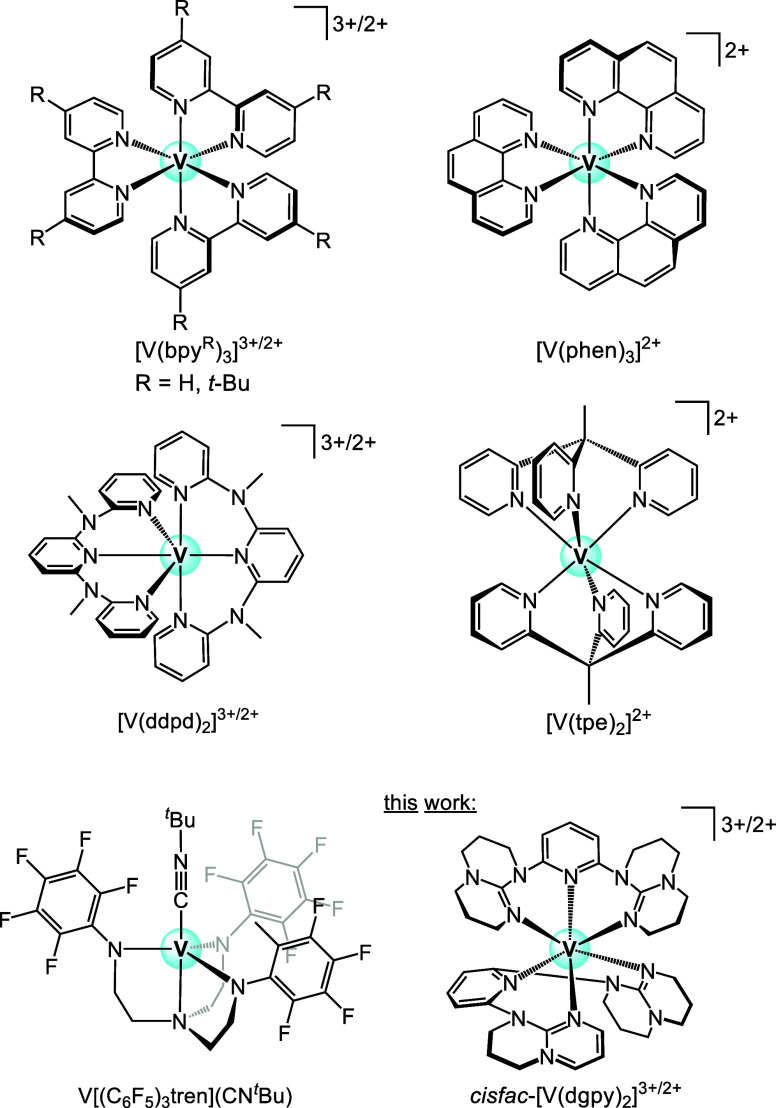
Selected vanadium­(III) and vanadium­(II) complexes.^1–6,11,12^

However, a large ligand field splitting alone is insufficient. Although 2,2′-bipyridine (bpy) and 9,10-phenanthroline (phen) are strong-field ligands, classical bpy and phen vanadium­(II) complexes (Chart 1) are nonemissive with excited state lifetimes of 0.43 and 1.6 ns, respectively.^1–3^ The absence of measurable radiative decay is due to a very low-lying excited state (<0.8 eV) that forms by mixing doublet metal-to-ligand charge-transfer (^2^MLCT) character into the SF states.^6,8^ This low-energy state efficiently decays nonradiatively according to the energy-gap law^39–42^ and energy transfer to CH vibrational overtones via an inductive-resonant mechanism (IRM) of nonradiative transitions.^43,44^ Hence, low-energy π* orbitals, as immanent to conjugated bpy and phen ligands, appear detrimental to vanadium­(II) SF emission.

The ^2^MLCT state can even become the ground state (ligand noninnocence) through ligand substitution with electron-withdrawing substituents.^9^ On the other hand, electronically isolated pyridines in the tripodal tpe ligands with π* orbitals at comparably high energy place the ^2^MLCT states at sufficiently high energy in [V­(tpe)_2_]^2+^, so that ^2^MLCT admixture to the doublet SF states is reduced. This energy tuning enables a low-energy luminescent excited state for [V­(tpe)_2_]^2+^ with a record lifetime of 760 ns at room temperature (Chart 1).^12^

An additional challenge for achieving SF emission from pseudo-octahedral vanadium­(III) complexes lies in the orbitally degenerate ^3^T_1_ ground state of d^2^ vanadium­(III) ions in an octahedral coordination geometry. This degeneracy is often significantly lifted, so that the energy gap to the lowest-energy SF state is further reduced.^4,7,45,46^ Again, energy-gap law^39–42^ and multiphonon energy transfer via IRM^43,44^ hamper SF luminescence in such systems. The ground-state splitting of the complexes VCl_3_(ddpd)^7^ and [V­(urea)_6_]­[ClO_4_]_3_ ^47^ amounts to 800/1100 cm^–1^ and 1400 cm^–1^, respectively. This appears to be small enough to allow weak emission to be observed in the solid state at 1102/1219/1256 nm and 1010/1175 nm, respectively.^7,46^ The hexapyridine complex [V­(ddpd)_2_]^3+^ displays SF luminescence in solution at 1100 nm, even though the ground-state splitting with ca. 1880/2690 cm^–1^ is quite large (Chart 1).^4^ The emission lifetime amounts to 790/8800 ns (93%/7%) in frozen solution at 77 K.^4^ The ground-state splitting of the d^2^ electron configuration in octahedral symmetry has been circumvented by turning to a pseudotrigonal-bipyramidal coordination geometry in the complex V­[(C_6_F_5_)_3_tren]­(CN^*t*^Bu) (Chart 1) with an orbitally nondegenerate ground state. This symmetry breaking enables SF luminescence in a frozen glass at 77 K or in the solid state with an excited state lifetime of 3 μs in the single crystal at 298 K.^5^

Here, we aim to tune the lifetimes and energies of the lowest-energy SF states of pseudo-octahedral vanadium­(II) and vanadium­(III) complexes using a mixed pyridine/guanidine ligand instead of classical pyridine-only ligands. The different donors should affect the excited-state mixing and the ground-state splitting and hence the energy gap and the dynamics between the SF and the ground state. We describe the synthesis and SC-XRD structures of new vanadium­(III) and vanadium­(II) complexes **[V­(dgpy)**_**2**_ **]** ^**3+/2+**^ with the mixed pyridine/guanidine ligand dgpy (2,6-diguanidylpyridine) with electron-poor and -rich subunits. The redox properties, gas-phase stabilities, as well as the ultrafast excited-state dynamics of both complexes were investigated by cyclic voltammetry, spectroelectrochemistry, electron-transfer dissociation (ETD), collision-induced dissociation (CID), and ultraviolet photodissociation (UVPD) mass spectrometry as well as ultrafast transient absorption (TA) spectroscopy, respectively. Density functional theory (DFT) as well as CASSCF-NEVPT2 calculations served to understand the experimental results. Subsequently, design criteria for optimized redox- and photoactive d^2^- and d^3^-vanadium­(III/II) complexes will be derived.

## Results and Discussion

### Synthesis and Characterization of *cisfac*-**[V­(dgpy)**_**2**_ **]­[OTf]**_**3**_ and *cisfac*-**[V­(dgpy)**_**2**_ **]­[OTf]**_**2**_

The ligand dgpy was obtained from 1,3,4,6,7,8-hexahydro-2*H*-pyrimido­[1,2-α]­pyrimidine and 2,6-dibromopyridine according to a literature procedure,^47^ separated from the KBr byproduct via extraction with benzene and carefully dried to avoid protonation of the basic guanidine sites (Figure S1), which would hamper complex formation. Vanadium­(III) triflate has been prepared before from VCl_3_ and HOTf,^3^ but a more convenient procedure has been developed starting from VCl_3_ and trimethylsilyl trifluoromethanesulfonate (TMSOTf) in CH_3_CN in the present study (Figures S2–S4). The absence of chloride and residual CH_3_CN in the green product V­(OTf)_3_ was carefully checked by reaction with AgPF_6_ (no formation of AgCl observed) and IR spectroscopy (absence of C≡N and C–H vibrational bands, Figure S3), respectively.

Coordination of two equivalents dgpy to vanadium­(III) triflate at room temperature in dry THF and recrystallization gave *cisfac* **-[V­(dgpy)**_**2**_ **]­[OTf]**_**3**_ as orange crystals in 20% yield (Scheme 1 and Figures S5 and S6). Reduction of *cisfac* **-[V­(dgpy)**_**2**_ **]­[OTf]**_**3**_ with KC_8_ ^48^ yielded *cisfac* **-[V­(dgpy)**_**2**_ **]­[OTf]**_**2**_ as black needles (CH_3_CN solvate) in 33% yield (Figures S7 and S8). The *cisfac* configuration of both complexes was confirmed by SC-XRD analyses (Figures 1 and S9 and Table S1). The V–N bond lengths in the d^3^-vanadium­(II) complex **[V­(dgpy)**_**2**_ **]** ^**2+**^ are very similar to 2.142(5)–2.190(5) Å, irrespective of the type of the N donor. In the d^2^-vanadium­(III) complex **[V­(dgpy)**_**2**_ **]** ^**3+**^, the V–N_pyridine_ bond lengths (2.109(3)/2.113(3) Å) are slightly longer than the V–N_guanidine_ bond lengths (2.068(3)–2.093(3) Å). The small bond expansion of <0.1 Å from vanadium­(III) to vanadium­(II) agrees with the lower charge and the population of essentially non-bonding d_π_ orbitals of vanadium­(II). The *cisfac* coordination mode of the dgpy ligands modifies the N–V–N angles, so that significant deviations from 90/180° result (**[V­(dgpy)**_**2**_ **]** ^**3+**^: 81–95°/175°; **[V­(dgpy)**_**2**_ **]** ^**2+**^: 77–96°/162–175°; Table S1). Interestingly, the *cisfac* isomer was also obtained as kinetic product for vanadium­(II) and the ddpd ligand, while the *mer* isomer was obtained at higher temperature.^11^ The dgpy ligand coordinates to manganese­(II) ions in both configurations,^49^ while other 3d metal ions appear to preferably form the *mer* isomers with dgpy.^49–53^ Thermal instability of dgpy precluded higher temperature during the synthesis to possibly form the *mer* isomer in the present case. Bond lengths and angles of both vanadium complexes are well reproduced by density functional theory (DFT) calculations (B3LYP, TZVPP, ZORA, CPCM (acetonitrile), D3BJ) (Table S1 and Figure S9). In addition, the experimental and calculated vibrational frequencies of the complex cations agree reasonably as well (Figures S5 and S7), suggesting that the bonding characteristics of the ^3^T_1_ and ^4^A_2_ ground states of the vanadium­(III) and vanadium­(II) complexes (see Figure S9 for spin densities) are sufficiently well described by the employed DFT method.

**Scheme 1:**
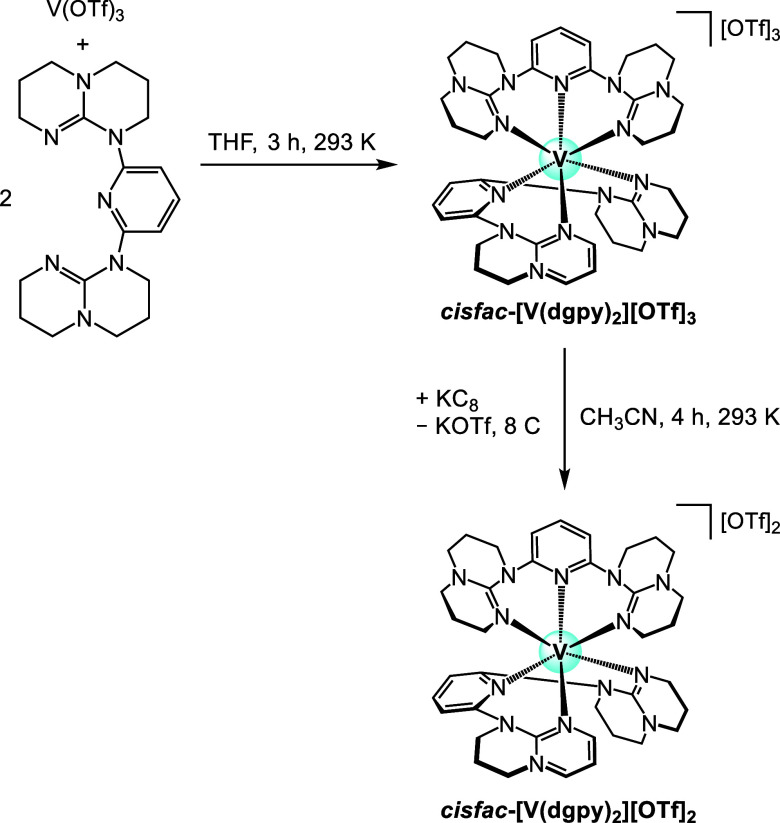
Syntheses of *cisfac* **-[V­(dgpy)**_**2**_ **]­[OTf]**_**3**_ and *cisfac* **-[V­(dgpy)**_**2**_ **]­[OTf]**_**2**_

**Figure 1:**
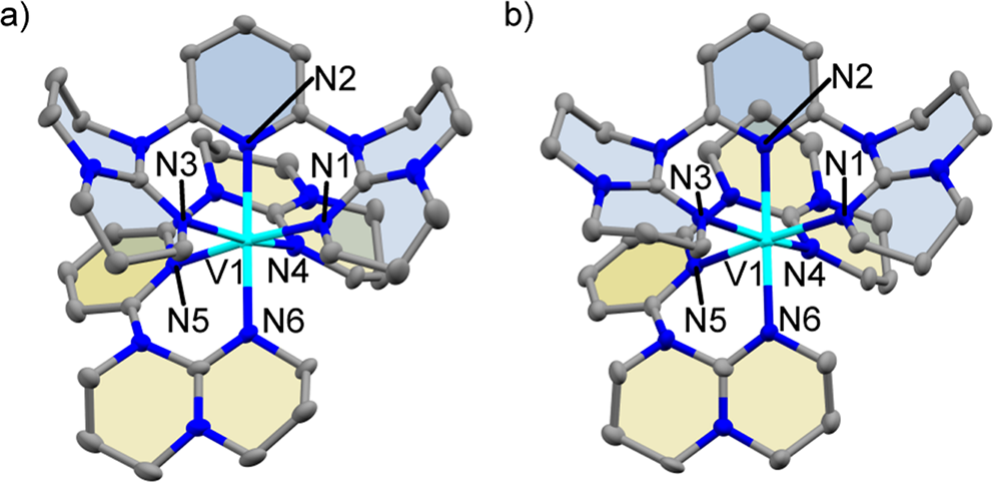
Molecular structures of the cations of (a) *cisfac* **-[V­(dgpy)**_**2**_ **]­[OTf]**_**3**_ and (b) *cisfac* **-[V­(dgpy)**_**2**_ **]­[OTf]**_**2**_ **×CH**_**3**_ **CN** determined by SC-XRD with thermal ellipsoids set to 50% probability. Hydrogen atoms, counterions, and solvent molecules are omitted. The rings of the different dgpy ligands are colored pale blue and pale yellow, respectively.

### Redox Chemistry of *cisfac*-**[V­(dgpy)**_**2**_ **]­[OTf]**_**3**_ and *cisfac* **-[V­(dgpy)**_**2**_ **]­[OTf]**_**2**_

As expected from the successful isolation of the complexes in both oxidation states and the retained coordination sphere, the vanadium­(III/II) redox couple at *E*_1/2_ = −1.26 V versus ferrocene is fully reversible in the cyclic voltammograms of *cisfac* **-[V­(dgpy)**_**2**_ **]­[OTf]**_**3**_ and *cisfac* **-[V­(dgpy)**_**2**_ **]­[OTf]**_**2**_ in CH_3_CN (Figures 2 and S10 and S11). In addition, a reversible wave is observed at *E*_1/2_ = +0.70 V. According to a DFT calculation on the d^1^ complex *cisfac* **-[V­(dgpy)**_**2**_ **]** ^**4+**^, this wave is assigned to the vanadium­(IV/III) couple (Figures 2 and S12 and Table S1). Ligand reduction is expected at very negative potentials and indeed lies outside our electrochemical window of CH_3_CN.

**Figure 2:**
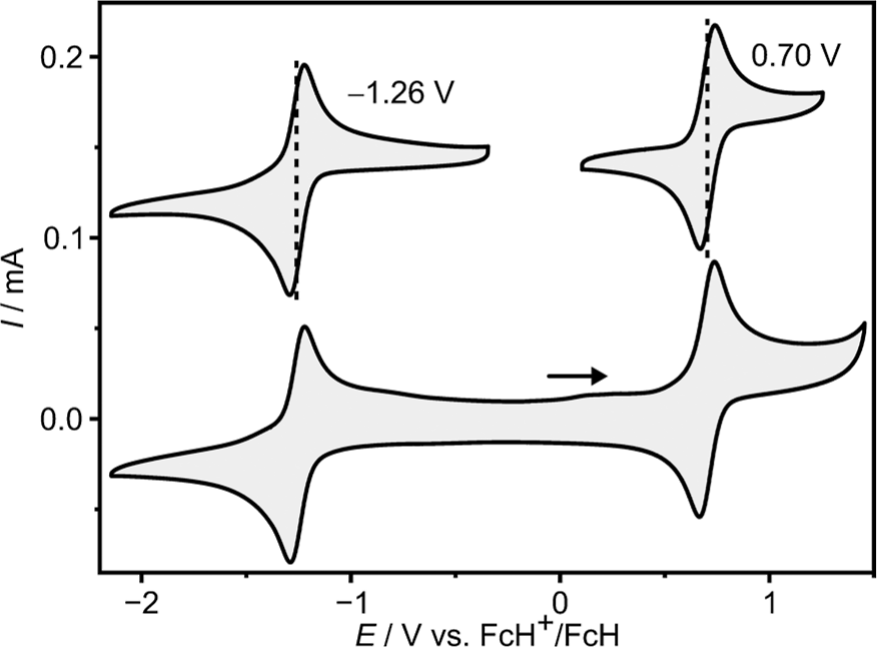
Cyclic voltammograms of *cisfac* **-[V­(dgpy)**_**2**_ **]­[OTf]**_**3**_ in CH_3_CN/[*n*-Bu_4_N]­[PF_6_]. Potentials given versus the ferrocenium/ferrocene couple.

Reductive spectroelectrochemistry of *cisfac* **-[V­(dgpy)**_**2**_ **]­[OTf]**_**3**_ in CH_3_CN confirms the full chemical reversibility of the *cisfac* **-[V­(dgpy)**_**2**_ **]** ^**3+/2+**^ couple showing an isosbestic point at 478 nm on longer time scales (Figure S13). Oxidative spectroelectrochemistry of *cisfac* **-[V­(dgpy)**_**2**_ **]­[OTf]**_**3**_ gave new absorption bands at 439 and 569 nm, but this process was only partially reversible on this time scale (Figure S14). It is conceivable that adventitious water in the spectroelectrochemical cell protonates and substitutes one of the basic guanidines, finally giving an oxido vanadium­(IV) complex.^2,12^

The fully reversible vanadium­(III/II) redox chemistry in the condensed phase prompted us to investigate the redox chemistry and the stabilities in the gas phase as well, complementing previous gas-phase studies of dgpy chromium­(III) and manganese­(IV/III) complexes.^53,54^ Electron transfer dissociation (ETD, Figure S15 and Table S2), collision-induced dissociation (CID, Figures 3a,d and S16 and Table S3), and ultraviolet photodissociation (UVPD, 220–400 nm, Figures 3b,c,e and S17 and S18) mass spectrometry experiments were conducted using vanadium­(III) and vanadium­(II) complex cations and their clusters with triflate counterions (see Supporting Information for a detailed discussion). In the CID experiments, dgpy ligand substitution by a triflate counterion prevails as major fragmentation channel both for vanadium­(III) and vanadium­(II) ions (Figure 3a,d). Interestingly, the cluster ion **{[V­(dgpy)**_**2**_ **]­[OTf]}** ^**2+**^ dissociates a triflate counterion (major path, Figure 3b) or a dgpy^•+^ radical cation (minor path, Figure 3b,e) giving **[V­(dgpy)**_**2**_ **]** ^**3+**^ and **{[V­(dgpy)]­[OTf]}** ^**+**^, respectively. In the UVPD experiments, the vanadium complexes and clusters deliver no or only relatively weak fragment ion intensities, respectively, suggesting a high photostability even in the UV spectral range. Under excitation with UV–C light, most pathways require more than one photon (multiphoton processes, Figure S18). Dissociation of a bidentate bpy ligand from [Ru­(bpy)_3_]^2+^ has been suggested to require two photons^55,56^ and a similar situation might be encountered here for the loss of the chelate ligand dgpy from metal centers.^54^ While the monocationic ion clusters **{[V­(dgpy)**_**2**_ **]­[OTf]}** ^**+**^ and **{[V­(dgpy)**_**2**_ **]­[OTf]**_**2**_ **}** ^**+**^ display identical CID/UVPD fragmentation patterns, the dicationic ion cluster **{[V­(dgpy)**_**2**_ **]­[OTf]}** ^**2+**^ dissociates a triflate anion giving **[V­(dgpy)**_**2**_ **]** ^**3+**^ under CID conditions but yields the dication **[V­(dgpy)**_**2**_ **]** ^**2+**^ under UVPD conditions (Figure 3b,c,e). The latter charge-transfer dissociation of the ion pair is induced by a single 265 nm photon (Figure S18). The electron affinity of the free OTf^•^ radical is estimated to be about 5 eV^57^ corresponding to a ca. 250 nm photon. Obviously, charge transfer from very high-energy states is possible within a contact ion pair, highlighting the stability of the **[V­(dgpy)**_**2**_ **]** ^**3+/2+**^ redox couple (Figure 2). The overall photostability of the complexes in both oxidation states is very high even under UV light excitation and absence of a solvent bath for energy dissipation.

**Figure 3:**
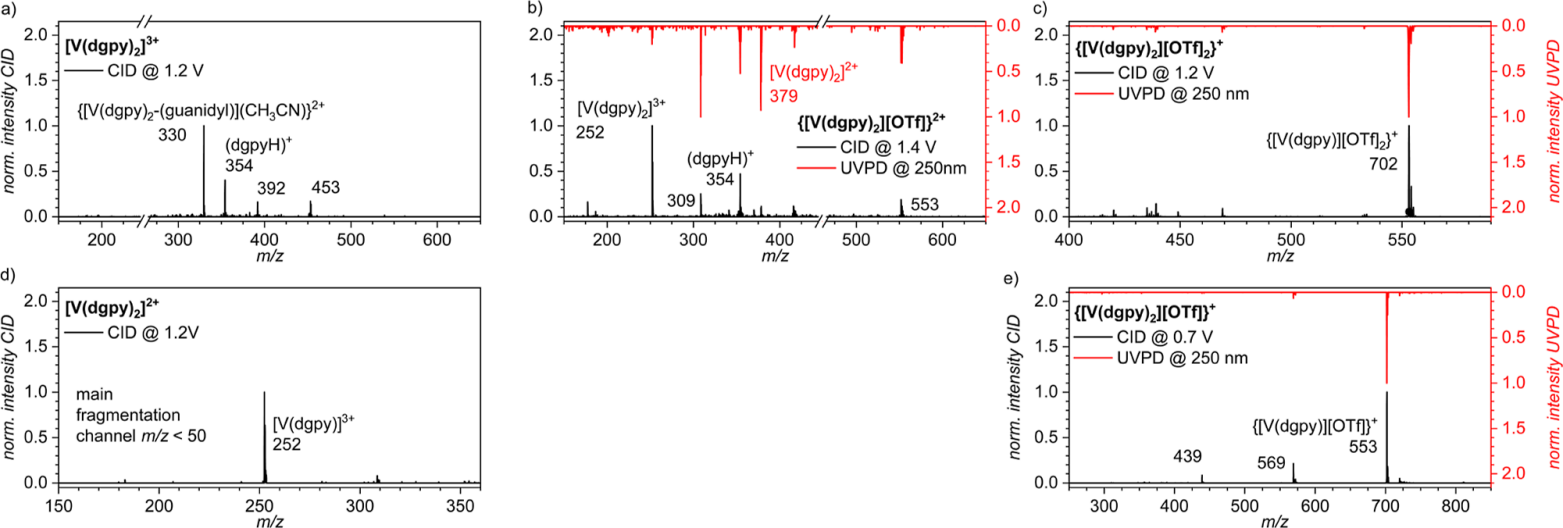
Normalized mass spectra (CID fragmentation, black) of (a) **[V­(dgpy)**_**2**_ **]** ^**3+**^, (b) {**[V­(dgpy)**_**2**_ **]­[OTf]}** ^**+**^, (c) {**[V­(dgpy)**_**2**_ **]­[OTf]**_**2**_ **}** ^**+**^, (d) **[V­(dgpy)**_**2**_ **]** ^**2+**^, and (e) {**[V­(dgpy)**_**2**_ **]­[OTf]}** ^**+**^ in CH_3_CN. Normalized mass spectra (UVPD fragmentation, red) at 250 nm excitation of (b) {**[V­(dgpy)**_**2**_ **]­[OTf]}** ^**2+**^, (c) {**[V­(dgpy)**_**2**_ **]­[OTf]**_**2**_ **}** ^**+**^ (weak ion intensity as a major channel is below the detection window, i.e., *m*/*z* <50), and (e) {**[V­(dgpy)**_**2**_ **]­[OTf]}** ^**+**^ in CH_3_CN. Complete assignments can be found in Table S3.

### Optical Spectroscopy of *cisfac*-**[V­(dgpy)**_**2**_ **]­[OTf]**_**3**_ and *cisfac*-**[V­(dgpy)**_**2**_ **]­[OTf]**_**2**_

Figure 4 depicts the optical absorption spectra of the two vanadium complexes in CH_3_CN. Beyond the intense ligand-centered and charge-transfer absorption bands in the UV spectral region that can lead to (multiphoton) photodissociation or electron transfer (in the gas phase, see above), the comparably high intensities of the absorption bands in the visible spectral region suggest that spin- and symmetry-allowed charge transfer transitions superimpose the in principle Laporte-forbidden^58^ ligand field transitions of the d^2^ and d^3^ complexes (in the pseudooctahedral local [VN_6_] symmetry). All of these low-energy excited states are photostable (in the gas phase, see above).

**Figure 4:**
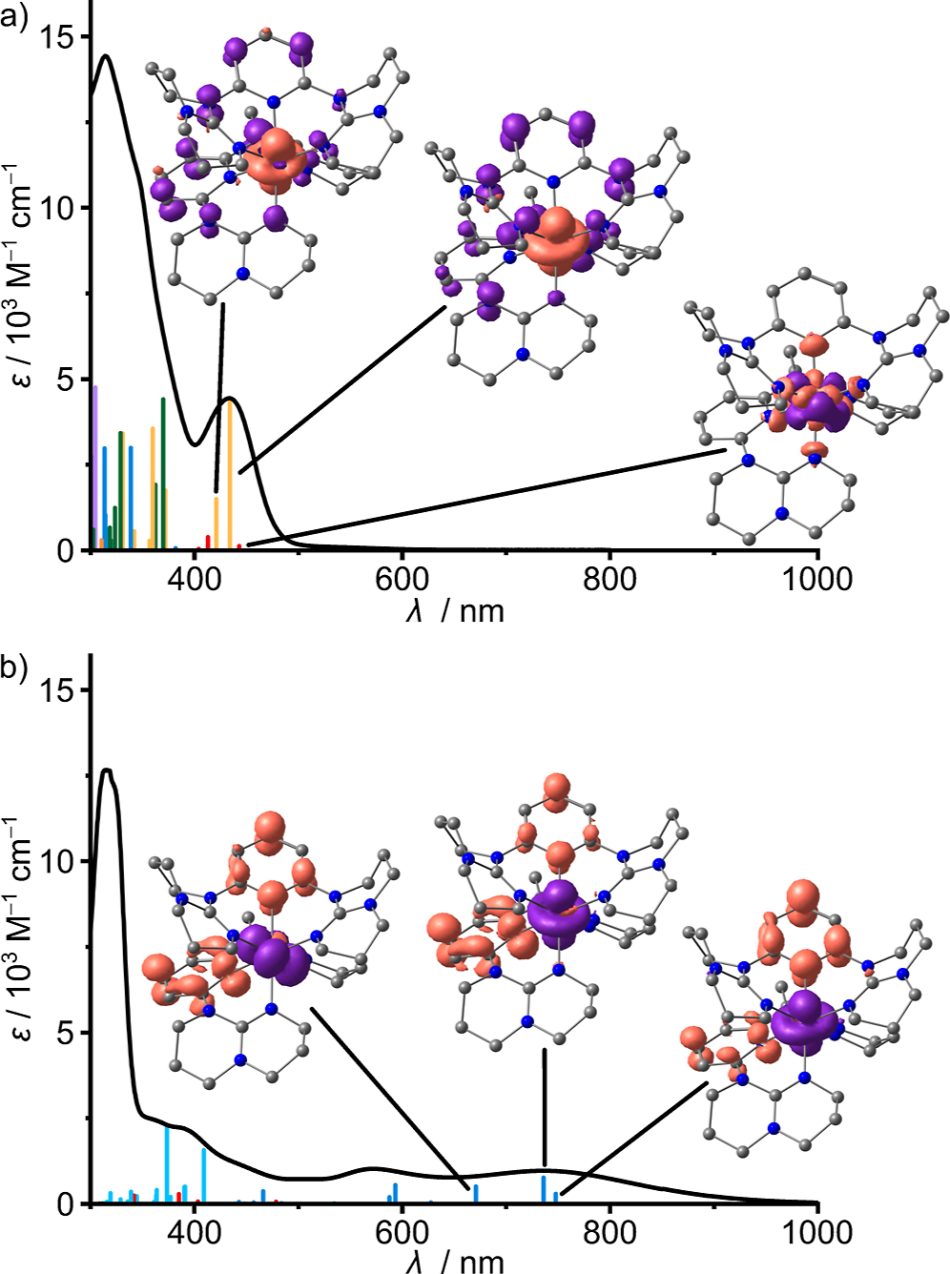
UV/vis/NIR absorption spectra (black) of (a) *cisfac* **-[V­(dgpy)**_**2**_ **]­[OTf]**_**3**_ and (b) *cisfac* **-[V­(dgpy)**_**2**_ **]­[OTf]**_**2**_ in CH_3_CN with TD-DFT-calculated oscillator strengths shown as vertical bars color-coded according to a charge-transfer number analysis (MC = red, LC = dark/light green (py/guanidine), MLCT = dark/light blue (V → py/guanidine), LMCT = dark/light orange (py/guanidine → V), ILCT = purple (guanidine→py)). Calculated transitions are shifted to higher energies in (a) and (b) by 883 and 1023 cm^–1^, respectively, to better match the experimental data. Electron density differences of the indicated selected Franck–Condon (FC) states of optimized *cisfac* **-[V­(dgpy)**_**2**_ **]** ^**n+**^ show electron density gain (orange) and depletion (purple) at an isosurface value of 0.003 au.

The ligand field transitions of **[V­(dgpy)**_**2**_ **]** ^**3+**^ are calculated by TD-DFT at 443, 413, and 404 nm (values shifted to higher energy by 883 cm^–1^) with weak intensities, while the intense absorption band at 434 nm (ε = 4400 M^–1^ cm^–1^) is largely composed of two allowed LMCT transitions calculated at 434 and 421 nm (Figures 4a and S19 and Table S4). The lowest-energy ^3^LMCT state (434 nm, 23,040 cm^–1^; energy of Franck–Condon (FC) state) is best described by electron density loss from the dgpy ligand (guanidine p_N_ orbitals and small contributions of the pyridine π orbital) and electron density gain of the vanadium center (Figure 4a and Table S4). The splitting of the orbitally degenerate ^3^T_1_ ground state (in octahedral symmetry) is calculated by DFT as 5017 and 5962 cm^–1^, respectively (Table S4).

The ligand field transitions of **[V­(dgpy)**_**2**_ **]** ^**2+**^ are calculated at 484, 479, and 466 nm with weak intensities, while the entire visible spectral region is dominated by intense MLCT absorption bands at 736 (ε = 1000 M^–1^ cm^–1^) and 571 nm (ε = 1000 M^–1^ cm^–1^) with calculated transitions at 748, 736, 671, 628, 593, 588, 535, 512, 457, 443, and 409 nm, respectively (values shifted to higher energy by 1023 cm^–1^) (Figures 4b and S20 and Table S5). The calculated lowest-energy ^4^MLCT state (748 nm, 13,370 cm^–1^; energy of FC state) is best described by electron density loss from the vanadium center and electron density gain of the pyridine donors (Figure 4b and Table S5). Clearly, the dominant CT character switches from LMCT to MLCT in the d^2^ and d^3^ dgpy vanadium complexes. Furthermore, the energy of the ^4^MLCT FC state of **[V­(dgpy)**_**2**_ **]** ^**2+**^ is much lower than that of the ^3^LMCT FC state of **[V­(dgpy)**_**2**_ **]** ^**3+**^. The energy orderings of the FC states will also affect the excited state dynamics. Both CT energies are only weakly dependent on solvent polarity (CH_3_CN, CH_2_Cl_2_, DMSO; Figures S19b and S20b), so that the excited-state dynamics was probed in CH_3_CN.

### Excited-State Dynamics of *cisfac*-**[V­(dgpy)**_**2**_ **]­[OTf]**_**3**_ and *cisfac*-**[V­(dgpy)**_**2**_ **]­[OTf]**_**2**_

To obtain a clear picture of the excited-state dynamics of the vanadium­(III) and vanadium­(II) complexes, possible population losses at early times after the excitation and the lifetime of the SF states, fs-TA spectra of *cisfac* **-[V­(dgpy)**_**2**_ **]­[OTf]**_**3**_ and *cisfac* **-[V­(dgpy)**_**2**_ **]­[OTf]**_**2**_ were recorded in CH_3_CN with pump pulses in the visible and probing in the UV to near-IR spectral region from 350 to 1600 nm (Figure 5).

**Figure 5:**
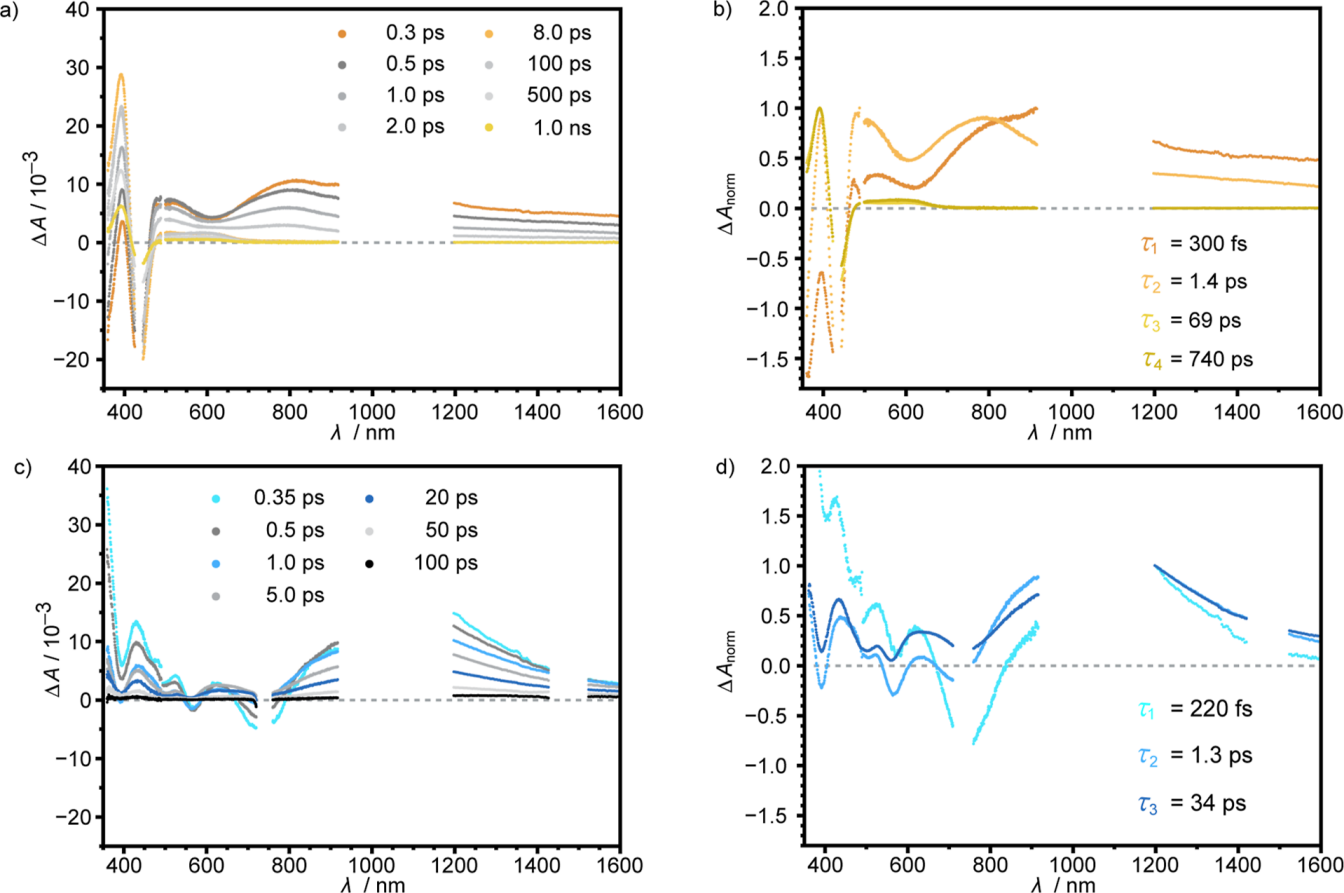
fs-Transient absorption spectra of (a) **[V­(dgpy)**_**2**_ **]­[OTf]**_**3**_ and (c) **[V­(dgpy)**_**2**_ **]­[OTf]**_**2**_ in CH_3_CN at 293 K after excitation at 435 and 740 nm, respectively. Corresponding evolution-associated difference spectra (EADS) of (b) **[V­(dgpy)**_**2**_ **]­[OTf]**_**3**_ and (d) **[V­(dgpy)**_**2**_ **]­[OTf]**_**2**_, respectively. The spectral regions in the vicinity of the respective excitation wavelengths 435/740 nm as well as the spectral range between 900 and 1200 nm are cutoff as those regions are superimposed with the scattered light of the pump pulse or the remaining fundamental (1030 nm) in the probe pulse, respectively.

fs-TA spectra obtained after excitation of *cisfac* **-[V­(dgpy)**_**2**_ **]­[OTf]**_**3**_ at 430 nm (mainly ^3^LMCT) in CH_3_CN are displayed in Figure 5a. Global analysis gave the evolution-associated difference spectra (EADSs) shown in Figure 5b and the associated time constants τ_1,2,3,4_ = 300 fs, 1.4 ps, 69 ps, and 740 ps, respectively. The first spectra with dominant excited-state absorption (ESA) bands above 600 nm correspond to a population of (hot) ^3^LMCT­(*n*) states which relax to the lowest-energy ^3^LMCT­(1) state by internal conversion (IC) and vibrational cooling (VC) with τ_1_ = 300 fs (Figure 6a,b). Subsequently, the electronic character changes significantly. The newly formed species possess only a weak ESA around 600 nm and a strong one below 400 nm with τ_2_ = 1.4 ps. We assign this transition to intersystem crossing (ISC) and VC from the ^3^LMCT­(1) state to high-energy singlet ligand field states ^1^MC’ (Figure 6a,b). IC and VC within the singlet manifold to the lowest-energy spin-flip ^1^MC states occur without strong changes of the ESA bands within τ_3_ = 69 ps (Figure 6a,b). The finally populated lowest-energy SF state has a lifetime of 740 ps (Figure 6a,b). This lifetime is too short to allow for SF luminescence, which is formally spin- and Laporte-forbidden^58^ and hence the radiative rate constant should be small. Clearly, this lowest-energy SF state decays efficiently via nonradiative pathways.

**Figure 6:**
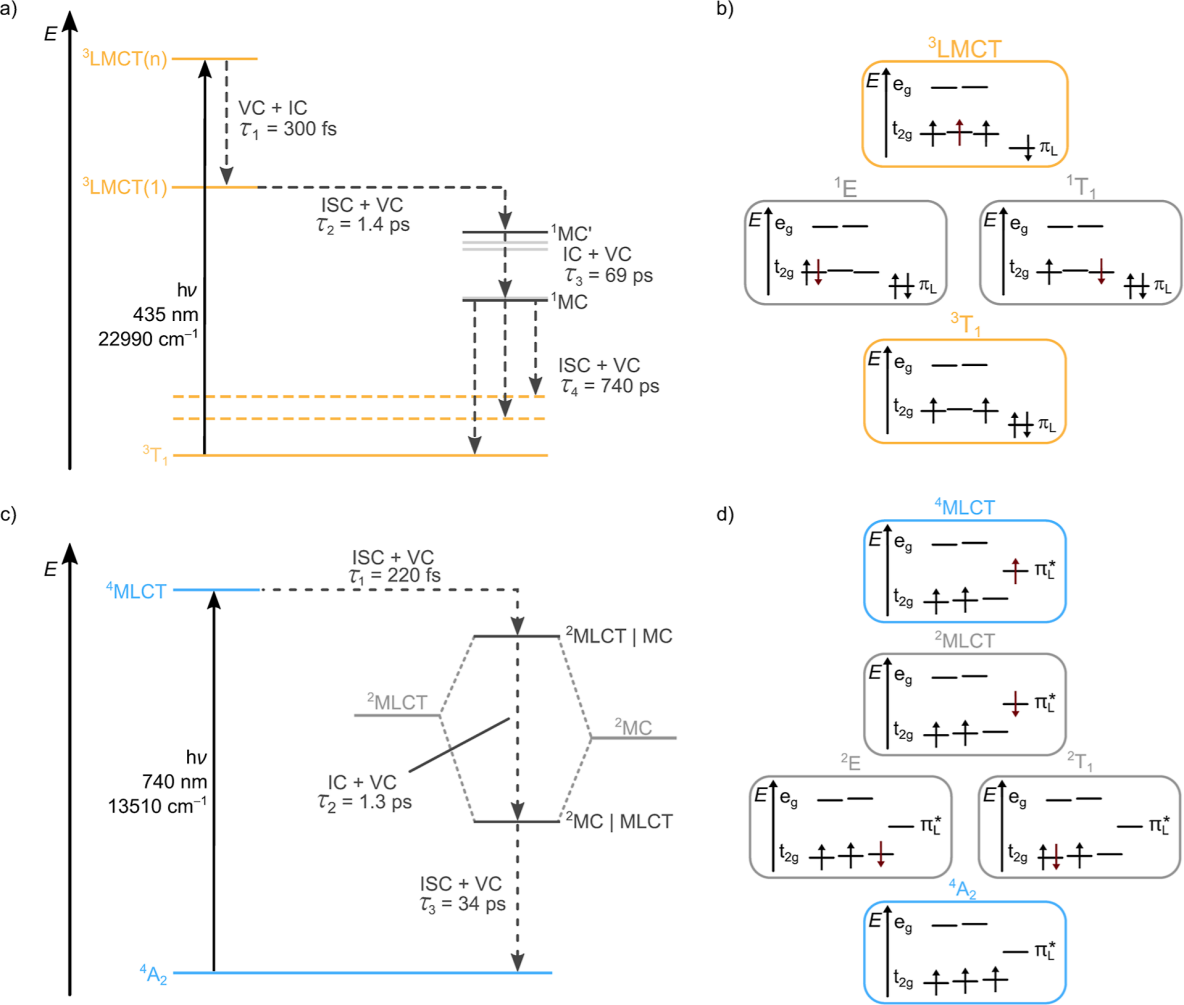
Kinetic models for the excited-state dynamics of (a) **[V­(dgpy)**_**2**_ **]­[OTf]**_**3**_ and (c) **[V­(dgpy)**_**2**_ **]­[OTf]**_**2**_. Time constants from the fs-TA experiments in CH_3_CN at 293 K. Energies of states beyond the FC states are arbitrarily set with some information from the quantum chemical calculations. The splitting of the ^3^T_1_ ground state and the energies of the metal-centered singlet states ^1^MC of **[V­(dgpy)**_**2**_ **]** ^**3+**^ are indicated by orange dashed and gray/black lines as obtained from CASSCF­(6,12)-NEVPT2 calculations at the DFT-optimized ground-state geometry. The (hypothetical) pure ^2^MLCT and ^2^MC states of **[V­(dgpy)**_**2**_ **]** ^**2+**^ are depicted in light gray for illustration purposes. Panels (b) and (d) illustrate the schematic orbital populations of the respective states (^1^MC = ^1^T_2_/^1^E; ^2^MC = ^2^T_1_/^2^E).

CASSCF­(6,12)-NEVPT2 calculations of the pure ligand field states of **[V­(dgpy)**_**2**_ **]** ^**3+**^ delivered the lowest-energy ^1^MC (^1^E/^1^T_2_) FC states at 9554 cm^–1^ and the ground-state splitting as 2308 and 3655 cm^–1^, respectively (Figure S21 and Tables S6 and S7). Hence, an extremely small energy gap of ν̃_SF_ = 5900 cm^–1^ to the highest level of the split ground state results, so that the energy gap law^39–42^ provides an efficient decay pathway (Figure 6a). In addition, nested, i.e., undistorted, excited states (weak coupling limit), as found for the lowest-energy singlet states according to unrestricted and restricted DFT optimizations (Figure S22 and Table S10), have been shown to decay via an IRM of nonradiative transitions, which involve high-energy oscillators close to the metal center (eqs 1, 2).^43,44^ The nonradiative rate constant depends on the energy-donor–acceptor distance d with d^–6^, an orientation factor κ, and the Förster-type spectral overlap integral SOI (eqs 1, 2).^43,44^ C–H stretching vibrations have been identified as deactivating modes for the respective [Cr­(dgpy)_2_]^3+^ complex with the 0 → 4 vibrational overtone of the dgpy methylene groups ν_CH_^4^ ≈11,000 cm^–1^ being resonant with the experimental SF emission energy of the chromium­(III) complex.^53^ In the present vanadium case, the 0 → 2 vibrational overtone of the methylene groups ν_CH_^2^ ≈ 5700 cm^–1^ is almost resonant with the calculated smallest energy gap ν̃_SF_ = 5900 cm^–1^. The much larger extinction coefficients ε_vib_ of lower overtones and hence a much larger SOI (eq 2), as compared to the chromium­(III) case with an orbitally nondegenerate ground state,^53^ provide another efficient nonradiative decay pathway via IRM.

1 $$k_{nr}\propto \kappa^{2}d^{-6}SOI$$

2 $$SOI=\int I_{norm}\left(\mathrm{\nu ̃}\right){\varepsilon_{vib}\left(\mathrm{\nu ̃}\right)\mathrm{\nu ̃}}^{-4}d\mathrm{\nu ̃}$$

The most decisive factor appears to be the very large ground-state splitting of the d^2^ ion. This ground-state splitting is already reflected in the splitting of the “t_2g_” orbitals with one d orbital being ca. 3140 cm^–1^ above the other two d orbitals (Table S6), which certainly arises from the different π-bonding capabilities of the pyridine and guanidine donors of the dgpy ligand. Consequently, vanadium­(III) complexes with similar ligand donor types and hence a smaller splitting of the ground state, e.g., [V­(ddpd)_2_]^3+^, are better candidates for SF emitters.^4,16^

Figure 5c shows the fs-TA spectra recorded after excitation of *cisfac* **-[V­(dgpy)**_**2**_ **]­[OTf]**_**2**_ at 740 nm (mainly ^4^MLCT states) in CH_3_CN. Global analysis gave the EADSs shown in Figure 5d and the associated time constants τ_1,2,3_ = 220 fs, 1.3 ps, and 34 ps, respectively. The first spectra with dominant ESA bands above 800 nm correspond to populated ^4^MLCT states. Ground-state bleach (GSB) appears around 570 and 735 nm with the former being superimposed by ESA bands. Interestingly, the overall pattern of the TA spectra and the EADSs remain comparably constant. This suggests that the electronic character does not fundamentally change and hence some MLCT character is retained during ISC/VC to the ^2^MLCT/^2^MC manifold (τ_1_ = 220 fs) and IC/VC (τ_2_ = 1.3 ps) processes within the doublet-state manifold (Figure 6c,d). The lifetime of τ_3_ = 34 ps of the finally populated doublet state is very short (Figure 6c,d). The prototypic pyridine vanadium­(II) complexes [V­(bpy)_3_]^2+^ and [V­(phen)_3_]^2+^ possess very small doublet excited-state energies due to ^2^MLCT admixture to the SF states.^6,8^ A similar situation is encountered here. Already the ^4^MLCT states are found at quite low energy with absorption bands tailing above 900 nm (Figure 4b). Hence, corresponding ^2^MLCT states will be located at even lower energy and can easily mix with the lowest-energy doublet SF states (^2^MC|^2^MLCT) leading to very low mixed doublet-state energies (Figure 6c,d). The energy of the lowest-energy pure SF state (^2^E/^2^T_1_) without CT mixing was calculated by CASSCF­(7,12)-NEVPT2 calculations at around 11,700 cm^–1^ (855 nm) (Figure S21 and Tables S8 and S9). The ^2^MLCT states are estimated to be lower in energy than the pure SF states and MLCT character will contribute to the lowest energy doublet state (Figure 6c,d). Indeed, the DFT-calculated lowest-energy doublet state with a very low energy of ca. 5930 cm^–1^ possesses some MLCT character with spin density located at one pyridine donor (Figure S22 and Table S10). The MLCT character induces a V–N_pyridine_ bond contraction of the reduced pyridine from 2.168 to 2.072 Å. This lowest-energy excited state efficiently decays nonradiatively as predicted by the energy gap law for distorted excited states (strong coupling limit).^39–42^ In addition, the lowest energy ^2^MLCT state might resonate with the 0 → 2 vibrational overtone ν_CH_^2^ ≈5700 cm^–1^ of the dgpy methylene groups.^53^ These combined nonradiative decay pathways dominate the excited-state decay of *cisfac* **-[V­(dgpy)**_**2**_ **]** ^**2+**^ resulting in the small excited-state lifetime τ_3_ = 34 ps.

The subnanosecond excited-state lifetimes of *cisfac* **-[V­(dgpy)**_**2**_ **]** ^**3+**^ and *cisfac* **-[V­(dgpy)**_**2**_ **]** ^**2+**^ do not favor spin-flip phosphorescence. Indeed, both vanadium complexes are nonemissive at 293 and 77 K, when excited into the respective lowest-energy absorption bands. The different donor properties provided by the dgpy ligands lead to a large ground-state splitting and hence a small energy gap to the lowest excited state in the d^2^ complex and to low-energy MLCT states in the d^3^ complex. Hence, future ligand design should consider uniform donors around vanadium­(III) to reduce the ground-state splitting and less electron-accepting ligands to increase the energy of MLCT states in vanadium­(II) complexes.

## Summary and Conclusions

The mixed-donor guanidine/pyridine ligand dgpy stabilizes vanadium in the oxidation states +II and +III in the pseudo-octahedral complexes *cisfac* **-[V­(dgpy)**_**2**_ **]** ^**3+/2+**^. In the gas phase, both complexes are highly UV/vis photostable requiring even more than one UV photon for photodissociation. In the dgpy vanadium­(III)–triflate ion pair **{[V­(dgpy)**_**2**_ **]­[OTf]}** ^**2+**^, charge transfer from the triflate counterion to the vanadium­(III) complex is induced by a single UV photon giving the vanadium­(II) complex. In CH_3_CN solution, the vanadium­(III/II) redox process is fully reversible as well on the cyclic voltammetry, spectroelectrochemistry, and chemical time scales. Excitation to the charge transfer absorption bands populates the lowest energy excited states after ultrafast intersystem crossing and vibrational relaxation. However, the lifetimes of the final excited states are on the subnanosecond time scale. Ground-state splitting and strong mixing with MLCT states strongly reduce the energy gap for the d^2^-vanadium­(III) and d^3^-vanadium­(II) complexes, respectively. In both cases, the low energy of the respective excited states favors nonradiative decay (energy gap law, inductive-resonant mechanism of nonradiative transitions) preventing luminescence. Hence, future ligand design should consider the stabilization of the different vanadium oxidation states, the overall complex symmetry (ground state splitting for d^2^), the reduction of MLCT mixing (d^3^), and the presence/absence of nearby X–H oscillators.

## Experimental Section

### General Procedures

The ligand dgpy was synthesized according to a modified literature procedure.^47^ All reagents were used as received from commercial suppliers (abcr, Acros Organics, Alfa Aesar, Thermo Fisher Scientific, Sigma-Aldrich, and TCI). Dry benzene was received from Sigma-Aldrich and used without further purification. VCl_3_ (anhydrous, 95%) and KC_8_ (anhydrous, 99.8%) were obtained from abcr. Solvents were dried by refluxing over sodium (diethyl ether) or calcium hydride (CH_3_CN), followed by distillation. [*n*-Bu_4_N]­[PF_6_] (≥99% for electrochemical analysis, Sigma-Aldrich) for electrochemical experiments was dried at 80 °C with reduced pressure (10^–3^ mbar) for 3 days and stored under argon. Syntheses and handling of air-sensitive compounds were either conducted using Schlenk techniques or a glovebox (UniLab/Mbraun—Ar 5.0; O_2_ <0.1 ppm; H_2_O <0.1 ppm).

**NMR spectra** were recorded on a Bruker Avance II 400 spectrometer at 400.13 MHz (^1^H) and 100 MHz (^13^C). Resonances for ^1^H and ^13^C­{^1^H} were reported in ppm versus the solvent signal as an internal standard [*d*_8_-THF (^1^H: δ = 3.58 ppm), (^13^C­{^1^H}: δ = 25.31 ppm)].^59^ (s) = singlet, (t) = triplet, (m) = multiplet.

**IR spectra** of air-sensitive samples (**[V­(dgpy)**_**2**_ **]­[OTf]**_**2**_, V­(OTf)_3_, and V­(CH_3_CN)_*n*_(OTf)_3_) were recorded with an Agilent Cary 630 FTIR spectrometer with a diamond ATR sampling accessory inside an argon-filled glovebox. The IR spectrum of **[V­(dgpy)**_**2**_ **]­[OTf]**_**3**_ was recorded with a Bruker Alpha II FTIR spectrometer equipped with an ATR unit containing a diamond crystal. Intensities were qualitatively indicated with sh (shoulder), weak (w), medium (m), and strong (s).

**Raman spectra** were measured on a Nicolet 5700 FT-IR spectrometer combined with a NXR 9650 FT-Raman Module equipped with a 1064 nm laser (laser power 20–1500 mW; resolution 2 cm^–1^; number of scans 1024–4098), a Microstage microscope, and a NXR Genie Ge-detector using single crystals or crystalline powders in glass capillaries under inert gas. The intensities are qualitatively indicated with weak (w), medium (m), and strong (s). **[V­(dgpy)**_**2**_ **]­[OTf]**_**2**_ was measured under continuous nitrogen cooling with a temperature of ca. 100 K due to light absorption of the sample (1064 nm) and subsequent heating of the sample.

**Electrochemical experiments** were carried out on a BioLogic SP-200 voltammetric analyzer. The measurements were performed in a glovebox, using dry CH_3_CN as the solvent. The working and counter electrodes consisted of platinum wire and 10 mM Ag/AgNO_3_ (100 mM [*n*-Bu_4_N]­[ClO_4_] in CH_3_CN) was used as the reference electrode. 100 mM [*n*-Bu_4_N]­[PF_6_] as supporting electrolyte and 1 mM of the sample were used. Cyclic voltammetry experiments were carried out at scan rates of 50–400 mV s^–1^. Potentials were referenced relative to the ferrocenium/ferrocene couple.

**UV/vis/NIR spectroelectrochemical experiments** were performed using a TSC 1600 Spectro cell from rhd instruments equipped with a platinum net working electrode (approximate path length 0.43 mm), a glassy carbon counter electrode, a silver wire as a pseudo reference electrode, a potentiostat Autolab IMP from Metrohm, and a J&M TIDAS S MMS UV/vis/NIR spectrometer with a Hamamatsu L10290 as an excitation source. Sample and cell preparation were done inside the glovebox, while the measurement was performed outside the glovebox.

**ESI** ^**+**^ **mass spectra** were measured on an Agilent 6545 QTOF-MS spectrometer in CH_3_CN.

**ESI** ^**+**^ **mass spectra for ETD and CID experiments** were recorded using a Bruker amZon ETD 3D Paul trap mass spectrometer, which is operated at room temperature. The sample solution of **[V­(dgpy)**_**2**_ **]­[OTf]**_**3**_ was prepared in dry and degassed CH_3_CN with concentrations of 10^–4^ to 10^–5^ M. The sample solution was continuously infused into the spray chamber using a syringe pump. Nitrogen was used as nebulizer gas and dry gas. The mass spectrometer is equipped with a negative chemical ionization (nCI) module to allow for electron transfer dissociation/reduction (ETD/ETR) experiments. Fluoranthene radical anions are prepared via chemical ionization. These are transferred into the ion trap where they can interact/react with the stored ions. For ETD experiments, the target ion was isolated and stored in the ion trap for 300 ms to allow for enough time for the electron transfer reaction. Collision-induced dissociation (CID) is used to induce fragmentation in a chosen precursor ion. Ions are stored in the 3D Paul trap (ion trap) of the mass spectrometer by a combination of DC voltage and radio frequency (RF). The trapped ions are accelerated by increasing the RF amplitude. Repeated collisions with the helium buffer gas transfer kinetic energy from the collisions into rotational–vibrational degrees of freedom. Internal vibrational redistribution leads to heating up of the stored ion and subsequently to fragmentation of the weakest bond.^60,61^ The full isotopic pattern of the respective vanadium species was isolated prior to CID experiments. CID breakdown curves are recorded by increasing the excitation amplitude (*E*_LAB_) from 0 to 3 V in 0.02 V steps. At each excitation amplitude, mass spectra were recorded for 300 ms. At each step, mass spectra were recorded for a total of 18 s and averaged afterward. Relative signal intensities are calculated according to eq 3, with *I*_abs_^*P*^ being the absolute intensity of the respective parent ion, and $I_{abs}^{F_{i}}$ the absolute fragment intensities.

3 $$I_{rel}^{P}\left(E_{LAB}\right)=\frac{\sum_{i}I_{abs}^{P_{i}}\left(E_{LAB}\right)}{\sum_{i}I_{abs}^{F_{i}}\left(E_{LAB}\right)+\sum_{i}I_{abs}^{P_{i}}\left(E_{LAB}\right)}$$

The instrument specific voltage applied as excitation amplitude, i.e., *E*_LAB_, was corrected in two steps to consider the different masses and different charge states *z* of the investigated ions.^62^

4 $$E_{COM}=E_{LAB}\times \frac{m_{He}}{m_{He}+m_{ion}}$$

5 $$E_{COMz}=E_{COM}\times z$$

The obtained intensities were fitted with a sigmoidal function (eq 6). The obtained *E*_COMz_^50%^ value is where half of the parent ions are fragmented and it can be used as a qualitative measure for the relative stabilities of ions of chemically similar structures.^62–64^

$$I_{fit}^{P}\left(E_{COMz}\right)=\frac{A}{1+e^{-B\left(E_{COMz}-E_{COMz}^{50\%}\right)}}$$ 6

The parameter *A* describes the fitted intensity of the parent ion, and the exponent is the slope of the sigmoidal curve.

**UV photodissociation measurements** were conducted using a modified Paul trap mass spectrometer (AmaZon Speed, Bruker Daltonics^65–67^ ), which allows the admittance of laser radiation into the ion trap. A UV/vis OPO laser system (EKSPLA NT 242 ^68^) was used to record the photon-induced fragmentation. The UV photodissociation (UVPD) spectra were obtained by trapping the isolated target ion for 150 ms, and laser pulses were directed through the trap overlapping with the ion cloud. The wavelength λ was varied stepwise with 2 nm per step from 220 to 400 nm. At each step, the energy of the laser pulses was set to *E*_pulse_ = 1 μJ with a statistical error of 3% at each data point. Mass spectra were recorded for 1 min and total fragment yields *Y*_total_ were calculated in dependency of the wavelength λ according to eq 7.

7 $$Y_{total}\left(\lambda \right)=\frac{\sum_{i}I_{abs}^{F_{i}}\left(\lambda \right)}{\sum_{i}I_{abs}^{F_{i}}\left(\lambda \right)+\sum_{i}I_{abs}^{P}\left(\lambda \right)}\times \frac{\lambda }{hcE_{pulse}}$$

With $\frac{\lambda }{hcE_{pulse}}$ normalizing the spectrum with regard to photon flux and pulse energy. The resulting total fragment yields for each point were averaged, and the corresponding standard deviations were used to calculate the error shown in Figures S17 and S18. Additionally, static measurements at λ = 265 and 320 nm were conducted as a function of pulse energy. The data at 265 nm is taken for 0.25–2.5 μJ pulse^–1^ in steps of 0.25 μJ pulse^–1^. The data is plotted in a double logarithmic fashion and fit with an allometric function *y* = *ax* ^*b*^ (Figure S18). The fitting parameter *b* gives an indication of the numbers of photons required to induce fragmentation. Processes with *b* ≈1 are named single-photon processes, while all processes with *b* significantly larger than one are multiphoton processes.^69,70^ The data at 320 nm shows the same qualitative behavior but a lower signal-to-noise due to the lower fragmentation yield.

The **elemental analyses** of air-stable **[V­(dgpy)**_**2**_ **]­[OTf]**_**3**_ and air-sensitive **[V­(dgpy)**_**2**_ **]­[OTf]**_**2**_ as well as dgpy were conducted by the microanalytical laboratory of the department of chemistry of the University of Mainz using an Elementar vario EL Cube and by the Mikroanalytisches Labor Kolbe, c/o Fraunhofer Institut UMSICHT, Oberhausen, Germany, respectively.

**UV/vis/NIR absorption spectra** were recorded with an Agilent Cary 5000 UV/vis/NIR spectrophotometer using 1.00 cm quartz cells equipped with a Schott valve to maintain an inert atmosphere.

**Luminescence spectra** were recorded with an FLS1000 spectrometer from Edinburgh Instruments equipped with the cooled photomultiplier detectors PMT-980 and N-G09 PMT-1700, together covering the spectral range between 200 and 1700 nm. For excitation, a xenon arc lamp Xe2 (450 W) was used.

**fs-Transient absorption experiments** were conducted using a Helios pump–probe setup from Ultrafast Systems paired with a regeneratively amplified 1030 nm laser (Pharos, Light Conversion, 1030 nm, <190 fs, 2 mJ). The effective laser repetition rate of 2 kHz was set via an internal pulse picker. A small portion of the 1030 nm fundamental was directed to the optical delay line and was subsequently used to generate broadband probe light by focusing the beam onto a sapphire for measurements in the vis/NIR range (450–900 nm). In the UV/vis spectral range (330–500 nm), the second harmonic was focused onto a second sapphire instead of the fundamental. In the NIR spectral region (1150–1600 nm), a YAG crystal was employed for the supercontinuum generation. The pump pulse was generated with an optical parametric amplifier (Apollo Y, Ultrafast Systems). The sample solutions were measured in a 1 mm quartz cuvette. To generate spectra that cover the entire spectral region from 350 to 1600 nm, the UV/vis and vis/NIR parts of the transient absorption spectra were recorded separately under identical conditions and were combined using the overlap of both data sets in the visible region (475–525 nm). The TA data in the NIR spectral region were also collected under identical conditions but were added unscaled to the combined TA spectra. Preprocessing of the data, including chirp and baseline correction, was performed using the Surface Xplorer 4.3.0 software from Ultrafast Systems. The open-source Python based data analysis tool KiMoPack 7.4.9 was employed for global analysis of the TA data.^71^

**Intensity data for crystal structure determination of [V­(dgpy)**_**2**_ **]­[OTf]**_**3**_ and **[V­(dgpy)**_**2**_ **]­[OTf]**_**2**_ **×CH**_**3**_ **CN** were collected with a STOE IPDS-2T or a STOE STADIVARI diffractometer from STOE & CIE GmbH with an Oxford cooling using Mo Kα radiation (λ = 0.71073 Å). The diffraction frames were integrated using the STOE X-Area^72^ software package and were corrected for absorption with STOE X-Red32 by Gaussian integration.^73^ The structures were solved with SHELXT^74^ and refined by the full-matrix method based on *F* ^2^ using SHELXL^75^ of the SHELX^76^ software package and the ShelXle^77^ graphical interface. All non-hydrogen atoms were refined anisotropically, while the positions of all hydrogen atoms were generated with appropriate geometric constraints and allowed to ride on their respective parent atoms with fixed isotropic thermal parameters. Crystallographic data for the structures reported in this paper have been deposited with the Cambridge Crystallographic Data Centre as supplementary publication no. CCDC-2513687–2513688.

### Crystallographic Data of **[V­(dgpy)**_**2**_ **]­[OTf]**_**3**_

C_41_H_54_F_9_N_14_O_9_S_3_V (1205.10); triclinic; *P*1̅, *a* = 12.831(3) Å, *b* = 12.995(3) Å, *c* = 17.196(3) Å, α = 86.59(3)°, β = 70.16(3)°, γ = 64.00(3)°; *V* = 2410.4(11) Å^3^, *Z* = 2; density (calculated) = 1.660 g cm^–3^; *T* = 120(2) K; μ = 0.442 mm^–1^; *F*(000) = 1244; crystal size 0.350 × 0.193 × 0.070 mm^3^; θ = 1.266° to 32.903°; −17 ≤ *h* ≤ 19, −19 ≤ *k* ≤ 19, −23 ≤ *l* ≤ 25; rfln collected = 56,550; rfln unique = 15,493 [*R*(int) = 0.0418]; completeness to θ = 25.242° = 99.9%; absorption correction from integration; max. and min transmission 0.971 and 0.857; data 15,493; restraints 1436; parameters 988; goodness-of-fit on *F* ^2^ = 1.108; final indices [*I* >2σ­(*I*)] *R*_1_ = 0.0916, w*R*_2_ = 0.2822; *R* indices (all data) *R*_1_ = 0.1171, w*R*_2_ = 0.2934; largest diff. peak and hole 3.809 and −2.295 e Å^–3^. The disorder of two triflate anions was described with a split atom model describing three split positions for each anion, requiring *same*, *simu*, and *isor* restraints.

### Crystallographic Data of **[V­(dgpy)**_**2**_ **]­[OTf]**_**2**_ **×CH**_**3**_ **CN**

C_42_H_57_F_6_N_15_O_6_S_2_V (1097.08); monoclinic; *P*2_1_/*n*, *a* = 23.983(5) Å, *b* = 9.0641(18) Å, *c* = 24.043(5) Å, β = 118.23(3)°; *V* = 4605 (2) Å^3^, *Z* = 4; density (calculated) = 1.582 g cm^–3^; *T* = 120(2) K; μ = 0.398 mm^–1^; *F*(000) = 2284; crystal size 0.700 × 0.061 × 0.043 mm^3^; θ = 2.445° to 27.499°; −31 ≤ *h* ≤ 31, −11 ≤ *k* ≤ 11, −30 ≤ *l* ≤ 31; rfln collected = 27,487; rfln unique = 10,566 [*R*(int) = 0.1328]; completeness to θ = 25.242° = 99.8%; semiempirical absorption correction from integrations; max. and min transmission 0.987 and 0.567; data 10,566; restraints 84; parameters 678; goodness-of-fit on *F* ^2^ = 1.150; final indices [*I* >2σ­(*I*)] *R*_1_ = 0.1153, w*R*_2_ = 0.1863; *R* indices (all data) *R*_1_ = 0.2363, w*R*_2_ = 0.2444; largest diff. peak and hole 0.560 and −0.577 e Å^–3^. The backbone of one guanidine moiety is disordered over two positions, which was modeled by using *rigu* and *simu* restraints.

**Density functional theory (DFT) and CASSCF-NEVPT2 calculations** were performed using the quantum computing suite ORCA 5.0.4.^78,79^ All calculations were computed on the Elwetritsch supercomputer at Rheinland-Pfälzische Technische Universität Kaiserslautern-Landau (RPTU) (hpc.rz.rptu.de). This is a member of the AHRP (Alliance for High Performance Computing Rhineland-Palatinate). Geometry optimizations were performed without symmetry constraints using Kohn–Sham orbitals DFT and the B3LYP functional^80–82^ in combination with the ZORA-def2-TZVPP basis as recontracted *Ahlrich’s* basis set def2-TZVPP^84^ with the auxiliary basis SARC/J^85–87^ as decontracted def2/J^88^ basis up to Kr to use the zeroth-order regular approximation to describe relativistic effects in all calculations (keyword *ZORA*).^89,90^ Tight convergence criteria were chosen for (TD-)­DFT calculations (keywords *tightscf* and *tightopt*). All DFT calculations make use of the resolution of identity (Split-RI-J) approach for the Coulomb term in combination with the chain-of-spheres approximation for the exchange term (keyword *RIJCOSX*).^91–94^ To account for solvent effects, a conductor-like screening model (keyword *CPCM* (acetonitrile)) modeling acetonitrile was used in all calculations.^95,96^ Atom-pairwise dispersion correction was performed with the *Becke*-*Johnson* damping scheme (keyword *D3BJ*).^97,98^ The spin states and multiplicities of the cations in their electronic ground states are as follows: d^1^-**[V­(dgpy)**_**2**_ **]** ^**4+**^ (*S* = 1/2, *M* = 2), d^2^-**[V­(dgpy)**_**2**_ **]** ^**3+**^ (*S* = 1, *M* = 3), and d^3^-**[V­(dgpy)**_**2**_ **]** ^**2+**^ (*S* = 3/2, *M* = 4). The SF states were optimized for d^2^-**[V­(dgpy)**_**2**_ **]** ^**3+**^ (*S* = 0, *M* = 1; both using restricted and unrestricted DFT) and d^3^-**[V­(dgpy)**_**2**_ **]** ^**2+**^ (*S* = 1/2, *M* = 2), respectively. A numerical frequency calculation confirmed that the optimized geometry corresponds to a minimum structure. Explicit counterions and/or solvent molecules were not taken into account. Fifty spin-allowed transitions were calculated by TD-DFT. The assignment of the state characters has been done dividing the molecule into three fragments (metal center, two pyridine, and four guanidine moieties) and calculating charge transfer (CT) numbers with TheoDORE.^99,100^

**CASSCF­(6,12)-SC-NEVPT2** calculations on **[V­(dgpy)**_**2**_ **]** ^**3+**^ were performed with the ZORA-def2-TZVPP basis as recontracted *Ahlrich’s* basis set def2-TZVPP^83^ in combination with the automatically generated auxiliary basis (keyword *AutoAux*).^101^ The zeroth-order regular approximation was used to describe relativistic effects in all calculations (keyword *ZORA*).^89,90^ The calculations were performed state averaged with ten triplet and 12 singlet roots, respectively. From CASSCF­(2,5) calculations, the σ-bonding counterparts (e_g_) to the metal-centered e_g_* orbitals (in idealized *O*_*h*_ symmetry) were identified by orbital inspection together with a second d shell (keyword *ExtOrbs doubleshell*). Ten triplet and 12 singlet roots were used to encompass all low energy metal-centered transitions (Table S7). The final CASSCF­(6,12) calculations were performed in conjunction with the strongly contracted N-electron valence perturbation theory to second order (keyword *PTMethod SC_NEVPT2*) in order to recover the missing dynamic correlation.^102–106^ To account for solvent effects, a conductor-like screening model (keyword *CPCM* (acetonitrile)) modeling acetonitrile was used in all calculations.^95,96^

**CASSCF­(7,12)-SC-NEVPT2** calculations on **[V­(dgpy)**_**2**_ **]** ^**2+**^ were performed with the ZORA-def2-TZVPP basis as recontracted *Ahlrich’s* basis set def2-TZVPP^83^ in combination with the automatically generated auxiliary basis (keyword *AutoAux*).^101^ The zeroth-order regular approximation was used to describe relativistic effects in all calculations (keyword *ZORA*).^89,90^ The calculations were performed state averaged with ten quartet and ten doublet roots, respectively. From CASSCF­(3,5) calculations, the σ-bonding counterparts (e_g_) to the metal-centered e_g_* orbitals (in idealized *O*_*h*_ symmetry) were identified by orbital inspection together with a second d shell (keyword *ExtOrbs doubleshell*). Ten quartet and ten doublet roots were used to encompass all low energy metal-centered transitions (Table S9). The final CASSCF­(7,12) calculations were performed in conjunction with the strongly contracted N-electron valence perturbation theory to second order (keyword *PTMethod SC_NEVPT2*) in order to recover the missing dynamic correlation.^102–106^ To account for solvent effects, a conductor-like screening model (keyword *CPCM* (acetonitrile)) modeling acetonitrile was used in all calculations.^95,96^

### Synthesis of dgpy

The ligand dgpy was synthesized according to a literature procedure from 2,6-dibromopyridine and 1,3,4,6,7,8-hexahydro-2*H*-pyrimido­[1,2-*a*]­pyrimidine using KO^*t*^Bu and Pd­(OAc)_2_/BINAP in toluene,^47^ using Schlenk techniques and dry solvents to avoid protonation.^53^ For purification, the product dgpy was extracted from the beige crude product containing KBr by Soxhlet extraction with dry benzene as the most convenient method giving a colorless solid. ^1^H NMR (400 MHz, *d*_8_-THF): δ/ppm = 7.36–7.34 (m, 1H), 7.16–7.12 (m, 2H), 3.86–3.83 (m, 4H), 3.32 (t, ^3^ *J* = 6.4, H), 3.17 (t, ^3^ *J* = 6.0 Hz, H), 3.11 (t, ^3^ *J* = 6.5 Hz, 4H), 1.98–1.92 (m, 4H), 1.82–1.76 (m, 4H). ^13^C­{^1^H } NMR (100 MHz, *d*_8_-THF): δ/ppm = 151.7 (s), 146.7 (s), 132.6 (s), 108.2 (s), 46.7 (d), 41.9 (s), 40.9 (s), 21.7 (s), 21.1 (s). Elemental analysis: calcd (%) for C_19_H_27_N_7_ (353.47 g mol^–1^): C, 64.56; H, 7.70; N, 27.74. Found (%): C, 64.57; H, 7.72; N, 27.69.

### Synthesis of V­(OTf)_3_

VCl_3_ (2.0 g, 12.7 mmol, 1.0 equiv) was suspended in acetonitrile (30 mL) and an excess of trimethylsilyl trifluoromethanesulfonate (TMSOTf) (50.0 g, 225.0 mmol, 17.7 equiv) was added. The mixture was heated under reflux for 7 days. After cooling to room temperature, the dark-green solution was filtered. The solution was concentrated to approximately 10 mL under reduced pressure, resulting in the precipitation of a turquoise solid. The solid was collected by filtration, washed with dry diethyl ether (3 × 15 mL), and dried overnight under reduced pressure giving turquoise V­(CH_3_CN)_*n*_(OTf)_3_ (4.96 g). The absence of chloride ions in this turquoise product was confirmed by a negative silver­(I)­chloride precipitation with silver­(I) hexafluorophosphate in dry acetonitrile. To remove the coordinated CH_3_CN at the vanadium­(III) center, a portion of the turquoise product (138.9 mg) was heated to 150 °C under reduced pressure overnight giving a pale-green solid (89.7 mg) that lacks vibrational bands of CH_3_CN in the IR spectrum. ESI^+^ MS (CH_3_CN): calcd. for [C_6_H_6_F_6_N_2_O_6_S_2_V]^+^ *m*/*z* = 430.9006, found: *m*/*z* (%) = 430.9000 (100%); calcd. for [C_8_H_9_F_6_N_3_O_6_S_2_V]^+^ = 471.9272, found: *m*/*z* = 471.9266 (5%). IR (ATR, turquoise V­(CH_3_CN)_*n*_(OTf)_3_ product): *ṽ* = 3010 (w, CH, CH_3_CN), 2946 (w, CH), 2328 (m, CN, CH_3_CN), 2300 (m, CN, CH_3_CN), 1350 (s), 1237 (s), 1180 (s), 1154 (s), 964 (s), 768 (m), 628 (s), 604 (s), 570 (m), 535 (m), 511 (s), 416 (s) cm^–1^. IR (ATR, pale-green V­(OTf)_3_ product): *ṽ* = 1359 (m), 1323 (w, sh), 1210 (s), 1083 (m), 993 (s), 947 (sh), 777 (w), 628 (s), 606 (m), 578 (sh), 503 (m), 449 (m) cm^–1^. UV/vis/NIR (CH_3_CN): λ (ε) = 488 (20), 611 nm (20 M^–1^ cm^–1^).

### Synthesis of *cisfac*-**[V­(dgpy)**_**2**_ **]­[OTf]**_**3**_

To avoid protonation of the strongly basic dgpy ligand under ambient conditions, which would hamper the complexation of vanadium­(III), the complex synthesis was conducted in a glovebox. V­(OTf)_3_ (100.1 mg, 0.20 mmol, 1.0 equiv) was dissolved in dry THF (5 mL). This solution was added to a solution of dgpy (148.3 mg, 0.42 mmol, 2.1 equiv) in dry THF (5 mL). After stirring for 3 h at 298 K, a brown precipitate formed. The solid was collected by filtration and washed with THF (3 × 2 mL). The solid was dissolved in CH_3_CN (4 mL). Dark-orange crystals of the **[V­(dgpy)**_**2**_ **]­[OTf]**_**3**_ were obtained by diffusion of diethyl ether into the CH_3_CN solution after several days (49.1 mg, 0.04 mmol, 20%). Crystals for SC-XRD analysis were obtained by crystallization from CH_3_CN/Et_2_O. Elemental analysis: calcd (%) for C_41_H_54_F_9_N_14_O_9_S_3_V (1205.08 g mol^–1^): C, 40.86; H, 4.52; N, 16.27. Found (%): C, 40.70; H, 4.86; N, 16.22. MS (CH_3_CN/ESI^+^): calcd for {[V­(dgpy)_2_]­[OTf]_2_}^+^: *m*/*z* = 1055.313, found: *m*/*z* (%) = 1055.312 (92); calcd for {[V­(dgpy)_2_]­[OTf]}^2+^: *m*/*z* = 453.180, found: *m*/*z* (%) = 453.180 (100); calcd for [V­(dgpy)_2_]^3+^: *m*/*z* = 252.469, found: *m*/*z* (%) = 252.469 (46). IR (ATR): *ṽ* = 3117 (w), 2951 (w), 2863 (w), 1567 (s), 1488 (w), 1461 (m), 1420 (m), 1376 (m), 1310 (m), 1259 (s, triflate), 1218 (m), 1204 (m), 1132 (s), 1055 (w), 1031 (w), 1026 (s, triflate), 949 (w), 916 (w), 880 (w), 851 (w), 794 (m), 751 (m), 732 (s), 725 (m), 633 (s), 612 (s), 571 (m, triflate), 565 (m), 514 (m, triflate), 434 (w), 408 (w) cm^–1^. Raman: *ṽ* = 2947 (w), 1593 (w), 1489 (w), 1457 (w), 1416 (m), 1373 (w), 1309 (w), 1279 (w), 1231 (w), 1197 (w), 1106 (w), 1070 (w), 1030 (w), 1008 (m, triflate), 933 (w), 881 (w), 741 (m, triflate), 665 (w), 556 (w), 523 (w), 442 (w) cm^–1^. UV/vis/NIR (CH_3_CN): λ (ε) = 434 (4400), 348 (10700), 314 (14400), 250 nm (30100 M^–1^ cm^–1^). CV (CH_3_CN/[*n*-Bu_4_N]­[PF_6_]): *E*_1/2_ = 0.70 V (rev.), −1.26 (rev.) V versus FcH^+^/FcH.

### Synthesis of *cisfac*-**[V­(dgpy)**_**2**_ **]­[OTf]**_**2**_

KC_8_ (14.3 mg, 0.105 mmol, 4.35 equiv) was suspended in acetonitrile (2 mL). An orange solution of **[V­(dgpy)**_**2**_ **]­[OTf]**_**3**_ (29.2 mg, 0.024 mmol, 1.00 equiv) in acetonitrile (2 mL) was added. After stirring for 4 h at 298 K, the solution turned black. The solution was filtered to remove the graphite. Crystals were obtained by slow diffusion of diethyl ether into the CH_3_CN solution. Black needles which were suitable for SC-XRD analysis were obtained (8.3 mg, 7.9 μmol, 33%) after several weeks. These crystals were dried under reduced pressure to remove solvents for further measurements. Elemental analysis (CH_3_CN-free sample): calcd (%) for C_40_H_54_F_6_N_14_O_6_S_2_V (1056.00 g mol^–1^): C, 45.50; H, 5.15; N, 18.57. Found (%): C, 45.36; H, 5.13; N, 18.51. MS (CH_3_CN/ESI^+^): calcd for {[V­(dgpy)_2_]­[OTf]_2_}^+^: *m*/*z* = 1055.313, found: *m*/*z* (%) = 1055.312 (25); calcd for {[V­(dgpy)_2_]­[OTf]}^+^: *m*/*z* = 906.361, found: *m*/*z* (%) = 906.362 (100); calcd for {[V­(dgpy)_2_]­[OTf]}^2+^: *m*/*z* = 453.180, found: *m*/*z* (%) = 453.181 (26); calcd for [V­(dgpy)_2_]^2+^: *m*/*z* = 378.704, found: *m*/*z* (%) = 378.705 (43); calcd for [V­(dgpy)_2_]^3+^: *m*/*z* = 252.469, found: *m*/*z* (%) = 252.470 (14). IR (ATR): *ṽ* = 2945 (w), 2859 (w), 1604 (s), 1582 (m), 1502 (m), 1426 (m), 1375 (m), 1323 (w), 1303 (w), 1273 (m, sh), 1260 (s, triflate), 1221 (m), 1195 (m), 1142 (m), 1062 (m), 1027 (s, triflate), 915 (w), 885 (w), 846 (w), 792 (m), 738 (m), 634 (w, triflate), 572 (m, triflate), 516 (m, triflate), 441 (w) cm^–1^. Raman: *ṽ* = 1616 (w), 1581 (s), 1502 (w), 1415 (w), 1064 (w), 1003 (s, triflate), 948 (w), 775 (w, triflate), 748 (w), 655 (m) cm^–1^. UV/vis/NIR (CH_3_CN): λ (ε) = 736 (1000), 571 (1000), 389 nm (2300 M^–1^ cm^–1^). CV (CH_3_CN/[*n*-Bu_4_N]­[PF_6_]): *E*_1/2_ = 0.72 V (rev.), −1.26 (rev.) V versus FcH^+^/FcH.

## Supplementary Material





## Data Availability

All the raw data from this manuscript have been uploaded to Zenodo and are freely available via the DOI 10.5281/zenodo.20050414.

## Notes

Cartesian coordinates of optimized geometries from quantum-chemical calculations (XYZ)
Analytical and spectroscopic data and results of quantum chemical calculations (PDF)
