# Supplementary material for: Redox Chemistry and Photophysics of the [V(dgpy) 2 ] 3+/2+ Redox Pair
Source: Inorg Chem. 2026 May 23;65(22):12271–83. doi: 10.1021/acs.inorgchem.6c00836 (PMC13250986; doi:10.1021/acs.inorgchem.6c00836)
Supplement: Supplementary file 2 [file ic6c00836_si_002.pdf]

Redox chemistry and photophysics of the $[V(dgpy)_2]^{3+/2+}$ redox pair

Alexandra König,[a] Marietta Goetz,[a] Robert Naumann,[a] Christoph Förster,[a] Jan Klett,[a] Maximilian E. Huber,[b] Philipp Weber,[b] Christoph Riehn,[b] Jennifer Meyer,[b] and Katja Heinze*[a]

[a] Department of Chemistry, Johannes Gutenberg University Mainz, Duesbergweg 10-14, 55128 Mainz (Germany), email katja.heinze@uni-mainz.de

[b] Fachbereich Chemie und Forschungszentrum OPTIMAS, Rheinland-Pfälzische Technische Universität Kaiserslautern-Landau (RPTU), Erwin-Schrödinger Str. 52, 67663 Kaiserslautern (Germany)

Supporting Information

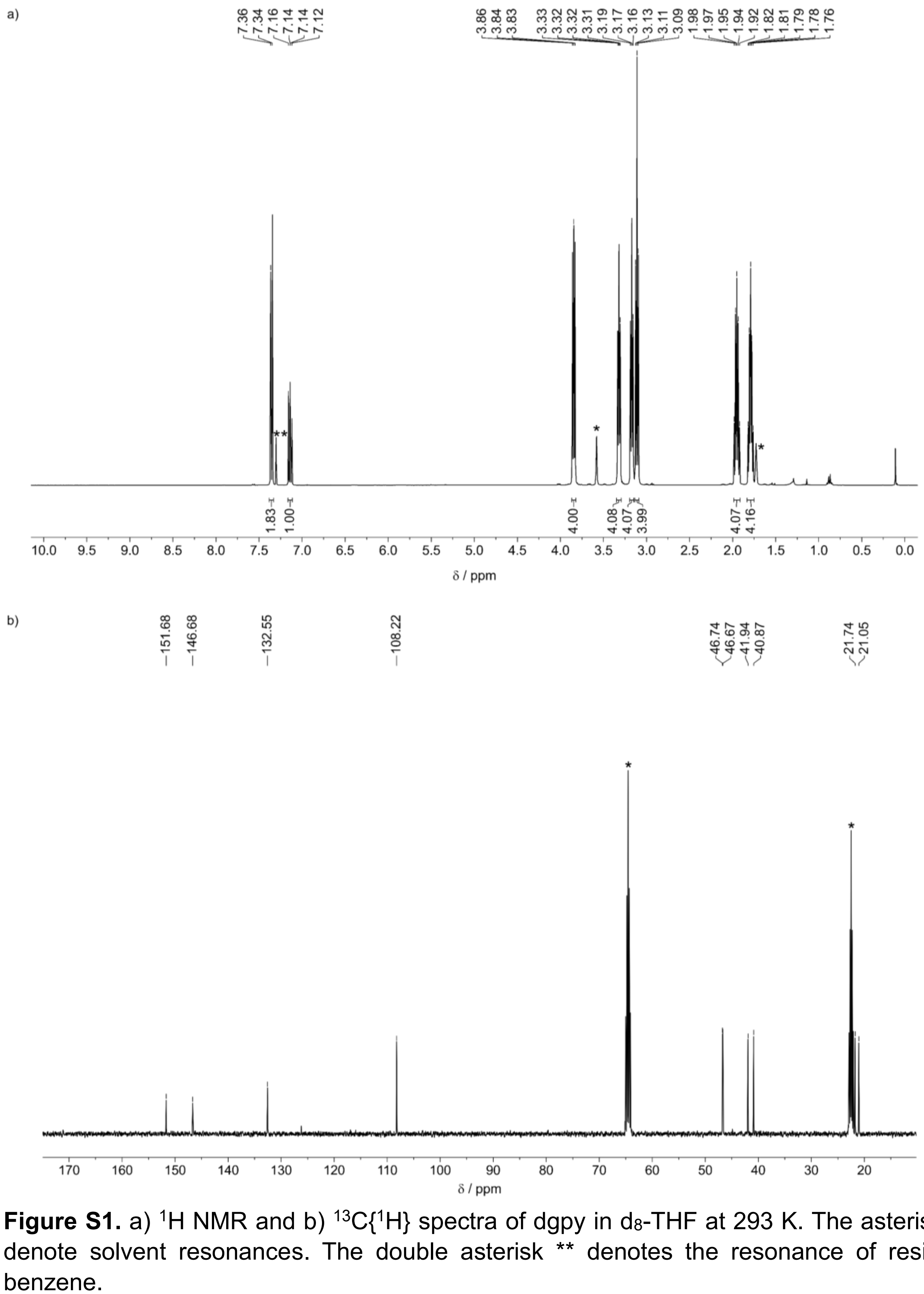

Figure S1. a) ^1H NMR and b) $^{13}C\{^1H\}$ spectra of dgpy in d_8-THF at 293 K. The asterisks * denote solvent resonances. The double asterisk ** denotes the resonance of residual benzene.

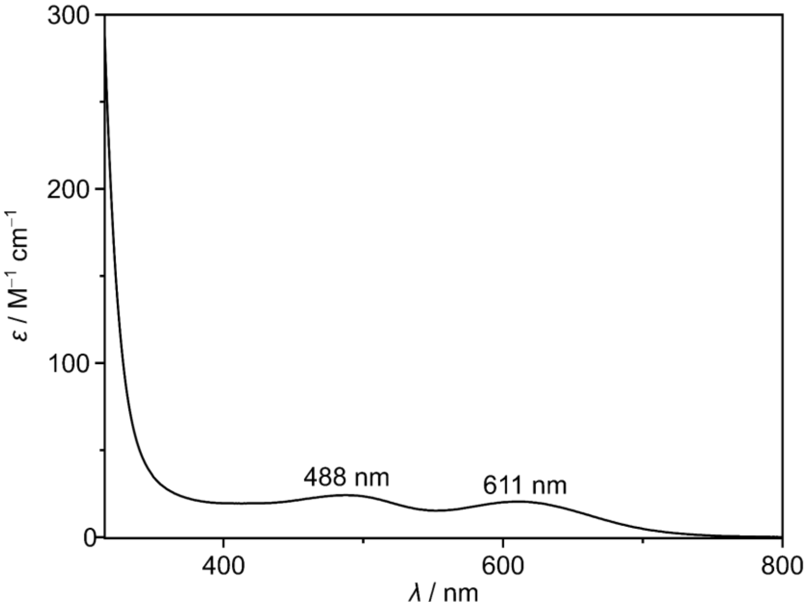

Figure S2. UV/vis/NIR absorption spectrum of $V(OTf)_3$ in CH_3CN at 293 K.

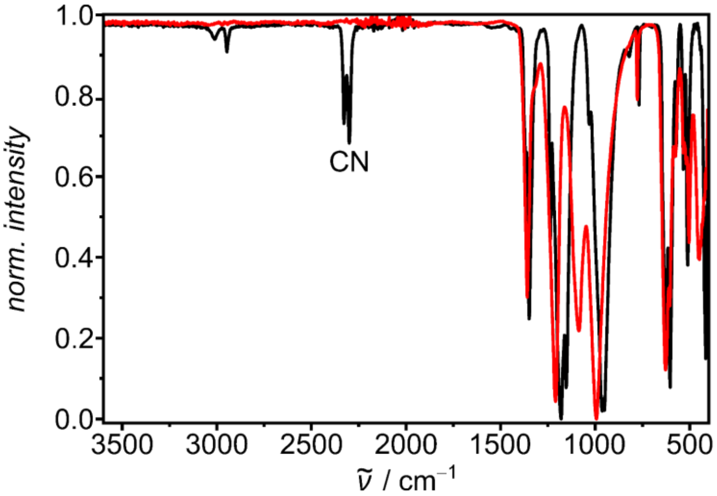

Figure S3. ATR-IR spectrum of $V(CH_3CN)_n(OTf)_3$ (black) and $V(OTf)_3$ (red) at 293 K.

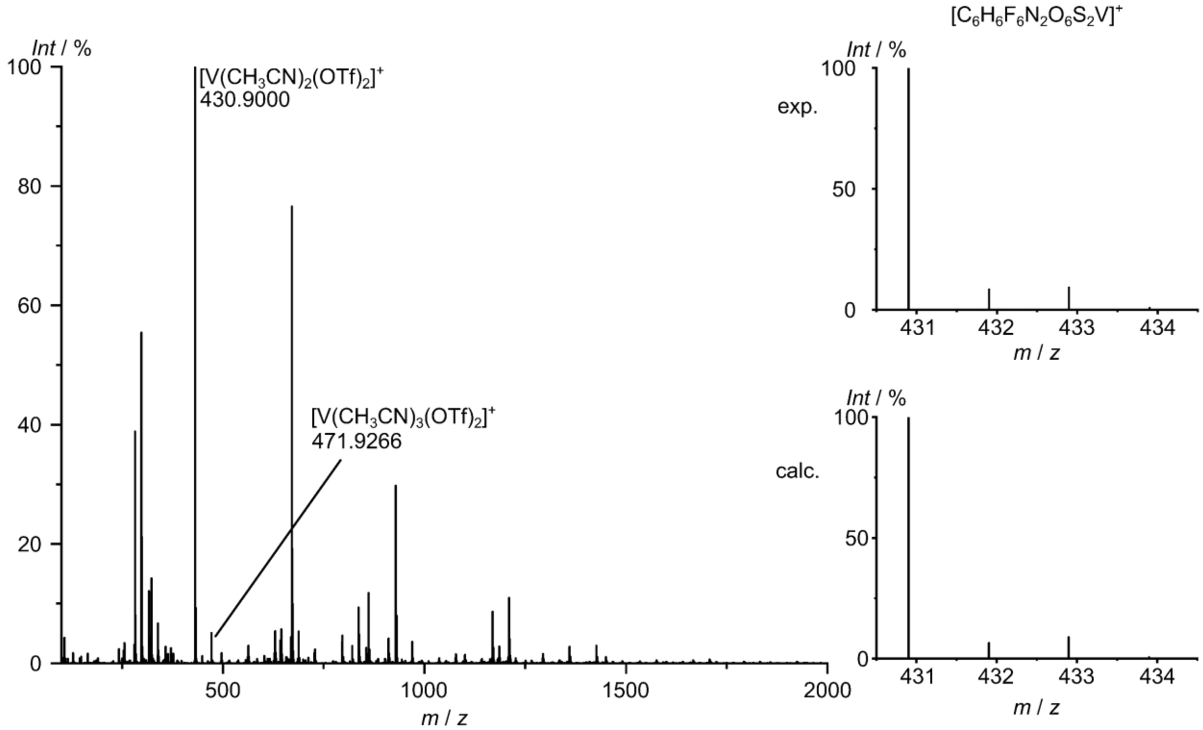

Figure S4. ESI$^+$ mass spectrum of $V(OTf)_3$ in CH_3CN. The insets show the calculated and experimental isotope distributions of the *m*/*z* peak of the cation $[V(CH_3CN)_2(OTf)_2]^+$.

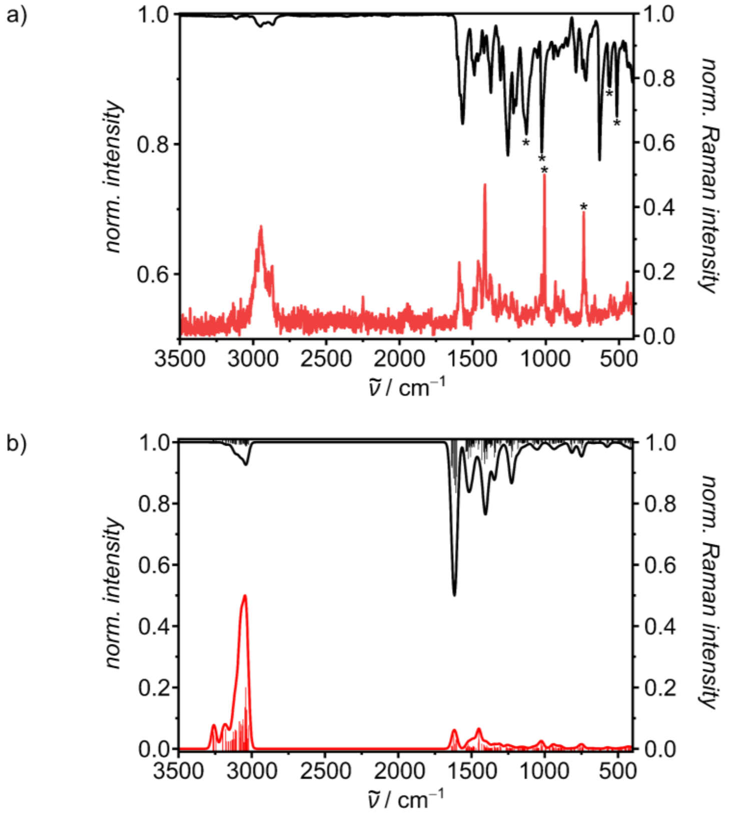

Figure S5. a) ATR-IR (black) and Raman (red) spectra of **[V(dgpy)$_2$][OTf]$_3$** at 293 K. b) DFT-calculated IR (black) and Raman (red) spectra of **[V(dgpy)$_2$]$^{3+}$** as well as the envelope band shape composed of individual Gaussian bands with a full width at half maximum FWHM = 35 cm^{-1}. No scaling was applied to the calculated frequencies. The asterisks * denote bands of the triflate counter ion.

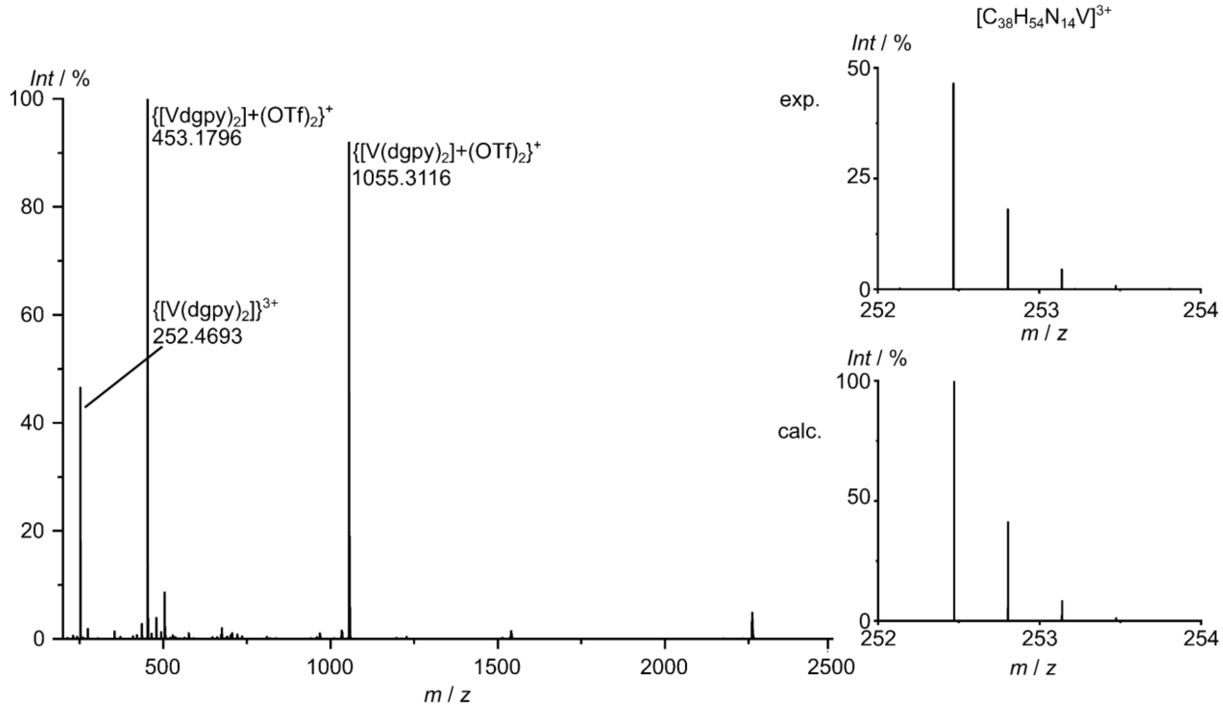

Figure S6. ESI$^+$ mass spectrum of **[V(dgpy)$_2$][OTf]$_3$** in CH_3CN. The insets show the calculated and experimental isotope distributions of the *m*/*z* peak of the trication **[V(dgpy)$_2$]$^{3+}$**.

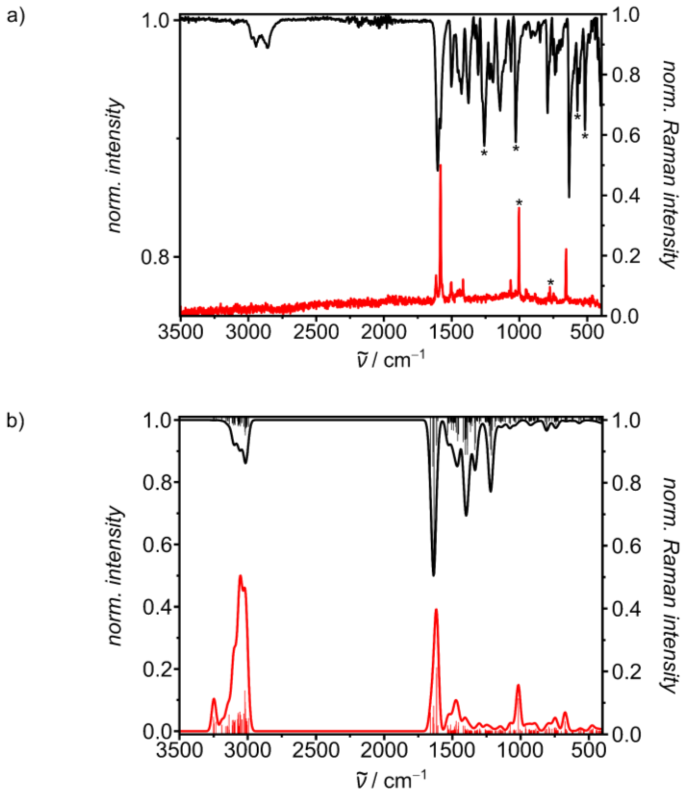

Figure S7. a) ATR-IR (black, 293 K) and Raman (red, ca. 100 K) spectra of **[V(dgpy)$_2$][OTf]$_2$**. b) DFT-calculated IR (black) and Raman (red) spectra of **[V(dgpy)$_2$]$^{2+}$** as well as the envelope band shape composed of individual Gaussian bands with a full width at half maximum FWHM = 35 cm^{-1}. No scaling was applied to the calculated frequencies. The asterisks * denote bands of the triflate counter ion.

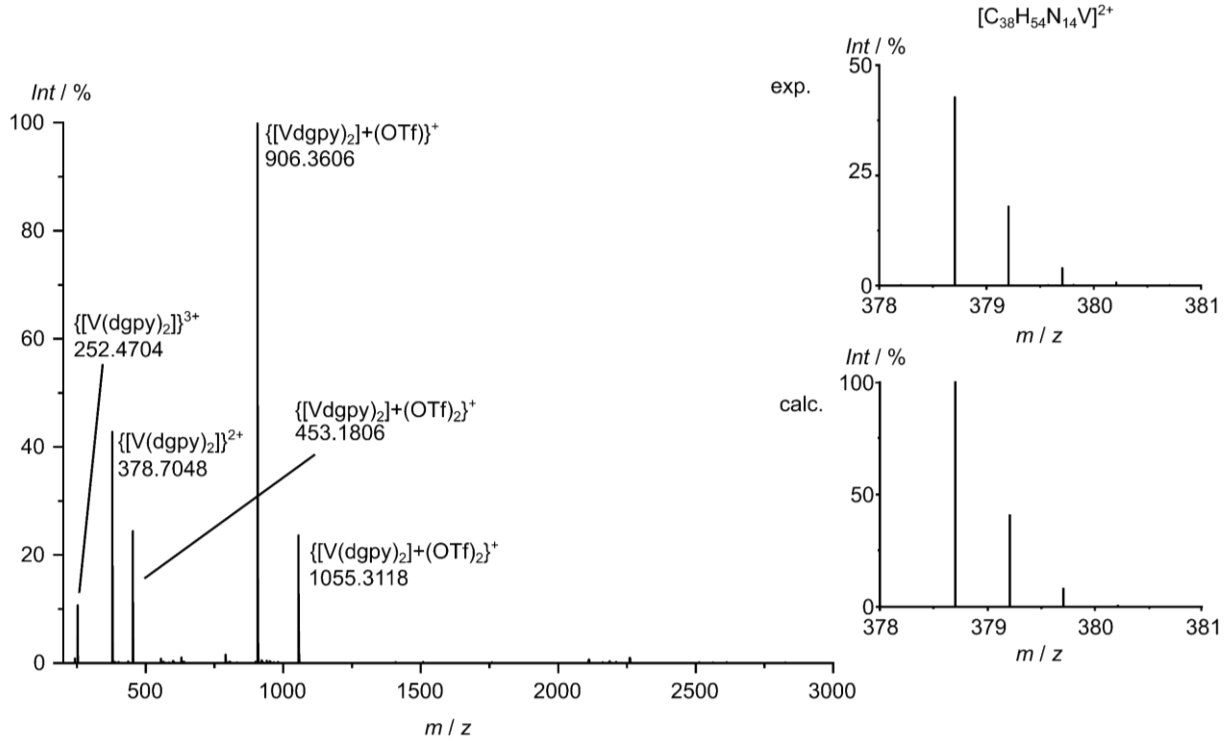

Figure S8. ESI$^+$ mass spectrum of **[V(dgpy)$_2$][OTf]$_2$** in CH_3CN. The insets show the calculated and experimental isotope distribution of the *m*/*z* peak of the di cation **[V(dgpy)$_2$]$^{2+}$**. As **[V(dgpy)$_2$]$^{2+}$** is oxidized to **[V(dgpy)$_2$]$^{3+}$** by oxygen, which is present in the spectrometer, this trication and its cluster ions are also observed in this experiment.

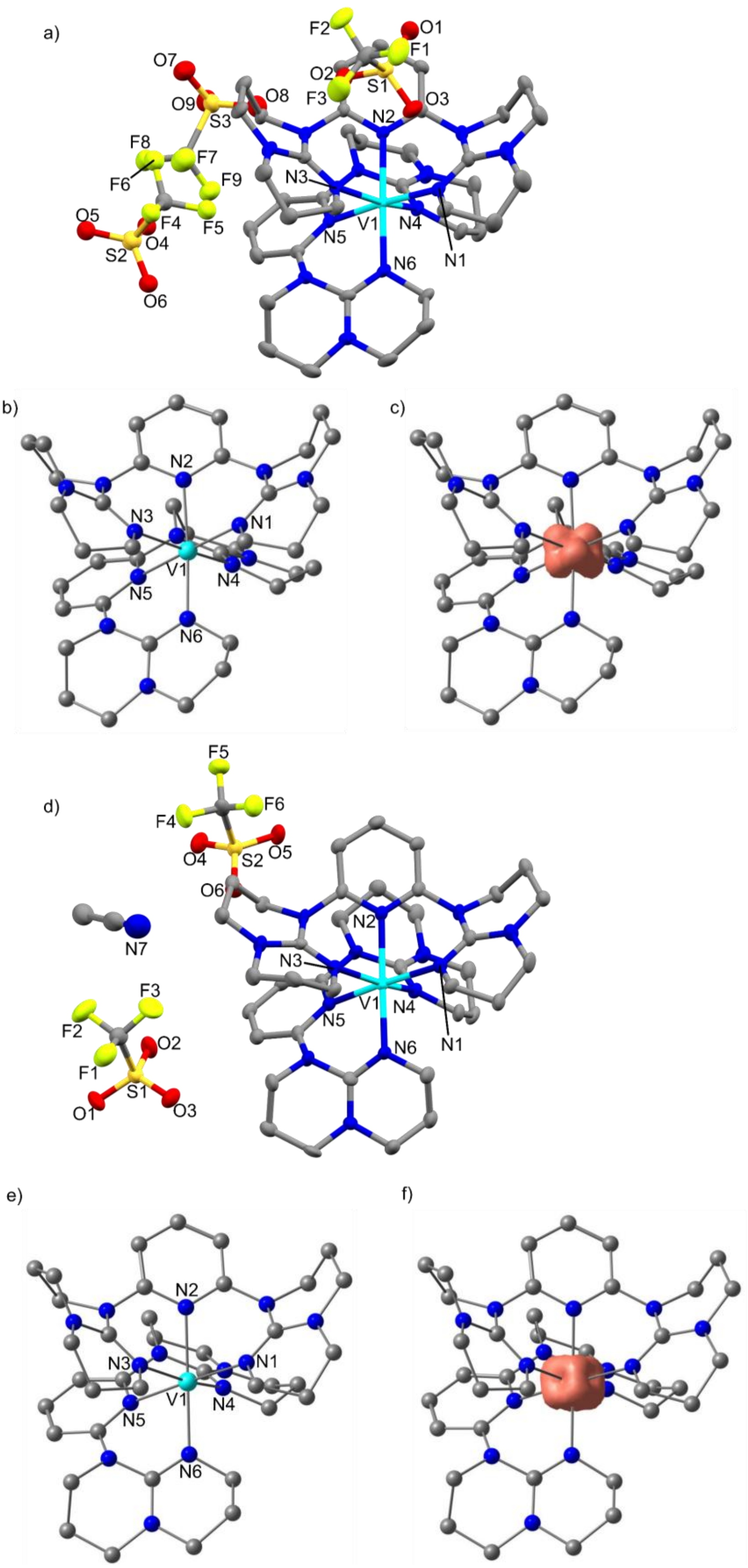

Figure S9. Crystal structures of a) **$[V(dgpy)_2][OTf]_3$** and d) **$[V(dgpy)_2][OTf]_2$**$\times CH_3CN$ with thermal ellipsoids set to 50 % probability. DFT-optimized geometries of b) **$[V(dgpy)_2]^{3+}$** and e) **$[V(dgpy)_2]^{2+}$** with atom umbering used in Table S1. Spin density plots of the ground state of c) **$[V(dgpy)_2]^{3+}$** and f) **$[V(dgpy)_2]^{2+}$**. α and β spin densities (orange/purple) are displayed with isosurface values of 0.03. Hydrogen atoms are omitted.

Table S1. Bond lengths [Å] and angles [deg] of **[V(dgpy)$_2$][OTf]$_3$** and **[V(dgpy)$_2$][OTf]$_2$xCH$_3$CN** from SC-XRD analyses and of **[V(dgpy)$_2$]$^{2+}$**, **[V(dgpy)$_2$]$^{3+}$** and **[V(dgpy)$_2$]$^{4+}$** in their electronic ground states obtained from DFT calculations.

	SC-XRD		DFT		
	**[V(dgpy)$_2$][OTf]$_3$**	**[V(dgpy)$_2$][OTf]$_2$** ×CH$_3$CN	**[V(dgpy)$_2$]$^{2+}$**	**[V(dgpy)$_2$]$^{3+}$**	**[V(dgpy)$_2$]$^{4+}$**
V1-N1	2.068(3)	2.155(6)	2.144	2.071	1.988
V1-N2	2.113(3)	2.146(6)	2.182	2.137	2.161
V1-N3	2.093(3)	2.171(7)	2.175	2.105	2.018
V1-N4	2.087(3)	2.195(6)	2.168	2.087	2.020
V1-N5	2.109(3)	2.156(6)	2.168	2.128	2.159
V1-N6	2.071(3)	2.146(6)	2.146	2.076	1.992
N1-V1-N2	82.59(12)	79.8(2)	79.78	81.65	83.47
N1-V1-N3	95.00(13)	95.9(2)	92.99	93.47	96.46
N2-V1-N3	80.62(12)	81.1(2)	81.32	82.51	79.78
N5-V1-N4	82.00(12)	77.1(2)	80.77	81.69	79.98
N6-V1-N4	94.99(13)	94.6(3)	93.19	93.61	96.51
N6-V1-N5	82.19(12)	82.6(2)	80.39	82.32	83.37
N1-V1-N5	175.19(12)	174.9(3)	177.29	175.63	178.60
N2-V1-N6	175.19(12)	174.1(2)	176.87	175.28	178.74
N3-V1-N4	175.34(12)	162.1(2)	165.28	169.24	156.16

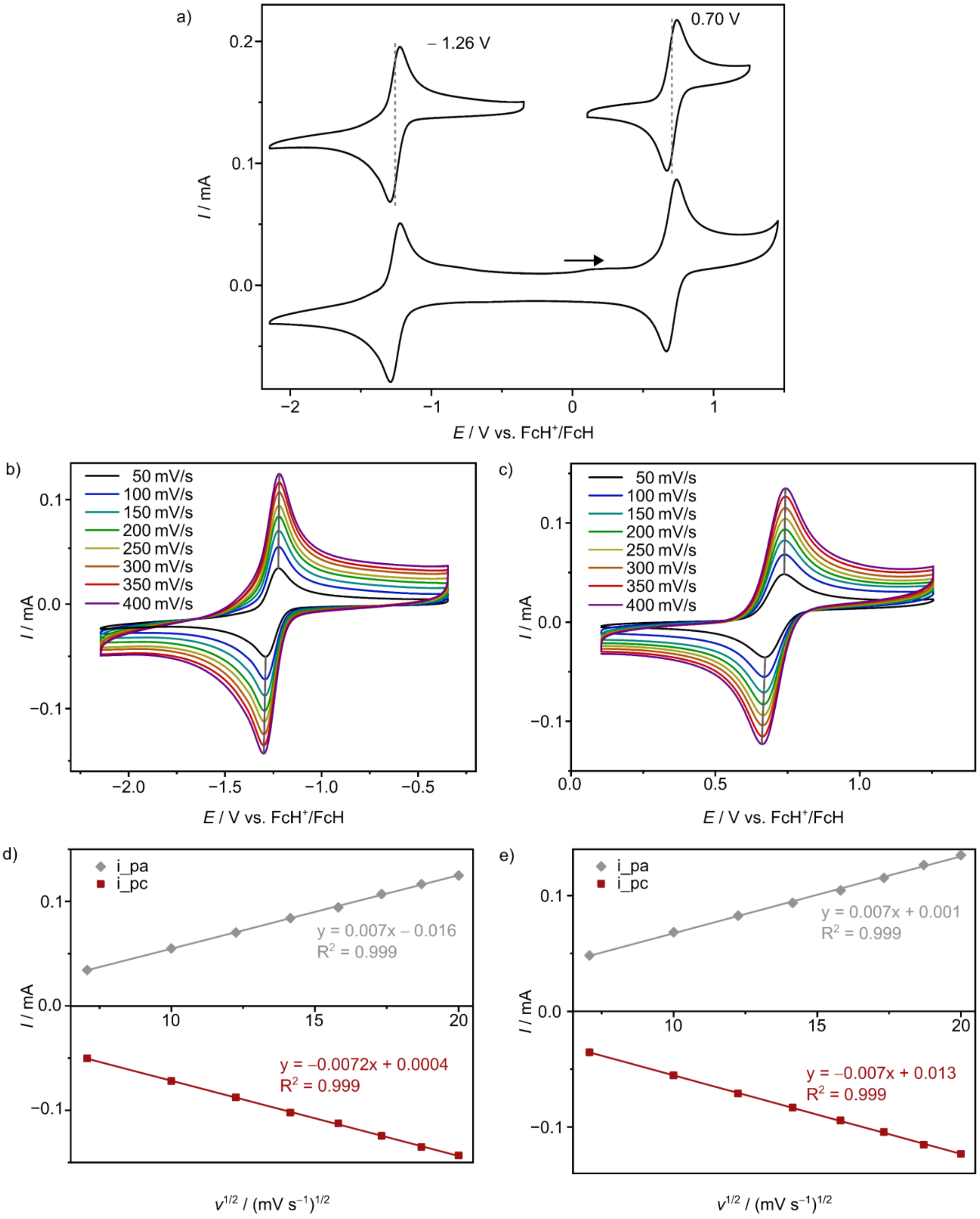

Figure S10. a) Full cyclic voltammogram of **[V(dgpy)$_2$][OTf]$_3$** in CH_3CN/[n-Bu_4N][PF_6] at 293 K; *E* vs. ferrocenium/ferrocene. b/c) Cyclic voltammograms at different scan rates of the **[V(dgpy)$_2$]$^{3+/2+}$** and **[V(dgpy)$_2$]$^{4+/3+}$** waves, respectively. d/e) Cathodic (red) and anodic (grey) peak currents at different scan rates plotted vs. the square root of the corresponding scan rate for **[V(dgpy)$_2$]$^{3+/2+}$** and **[V(dgpy)$_2$]$^{4+/3+}$** waves, respectively. Linear regression indicated.

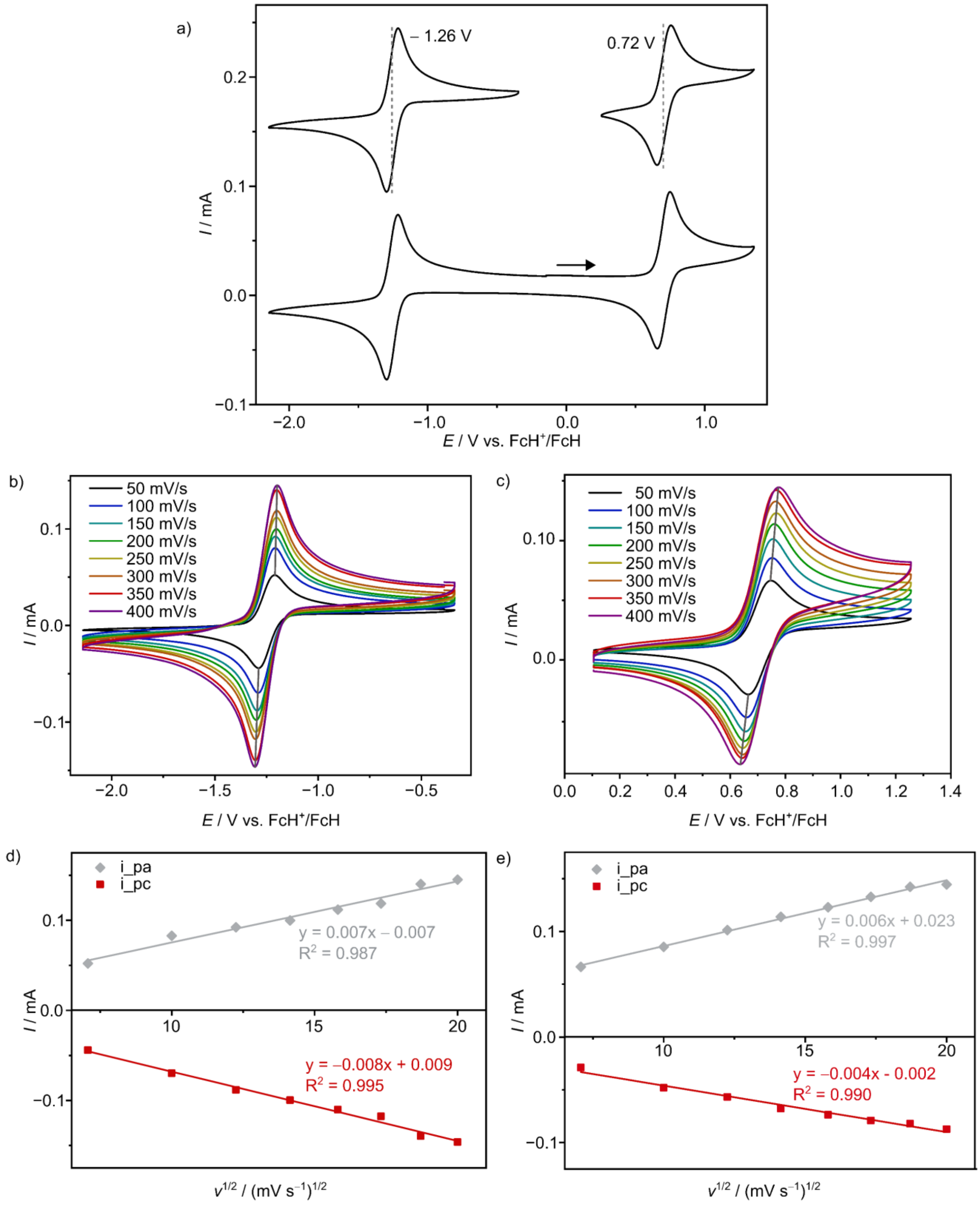

Figure S11. A) Full cyclic voltammogram of **[V(dgpy)$_2$][OTf]$_2$** in CH_3CN/[*n*-Bu_4N][PF_6] at 293 K; *E* vs. ferrocenium/ferrocene. b/c) Cyclic voltammograms at different scan rates of the **[V(dgpy)$_2$]$^{3+/2+}$** and **[V(dgpy)$_2$]$^{4+/3+}$** waves, respectively. d/e) Cathodic (red) and anodic (grey) peak currents at different scan rates plotted vs. the square root of the corresponding scan rate for **[V(dgpy)$_2$]$^{3+/2+}$** and **[V(dgpy)$_2$]$^{4+/3+}$** waves, respectively. Linear regression indicated.

a) b)

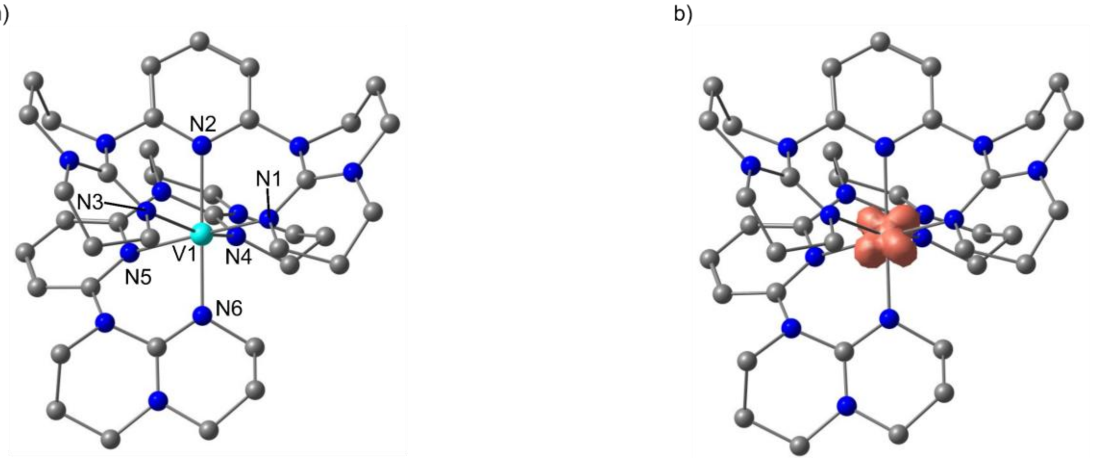

Figure S12. a) DFT-optimized geometry of **$[V(dgpy)_2]^{4+}$** with atom numbering used in Table S1 and b) spin density plot of DFT-optimized **$[V(dgpy)_2]^{4+}$**. Hydrogen atoms are omitted. α and β spin densities (orange/purple) are displayed with isosurface values of 0.03.

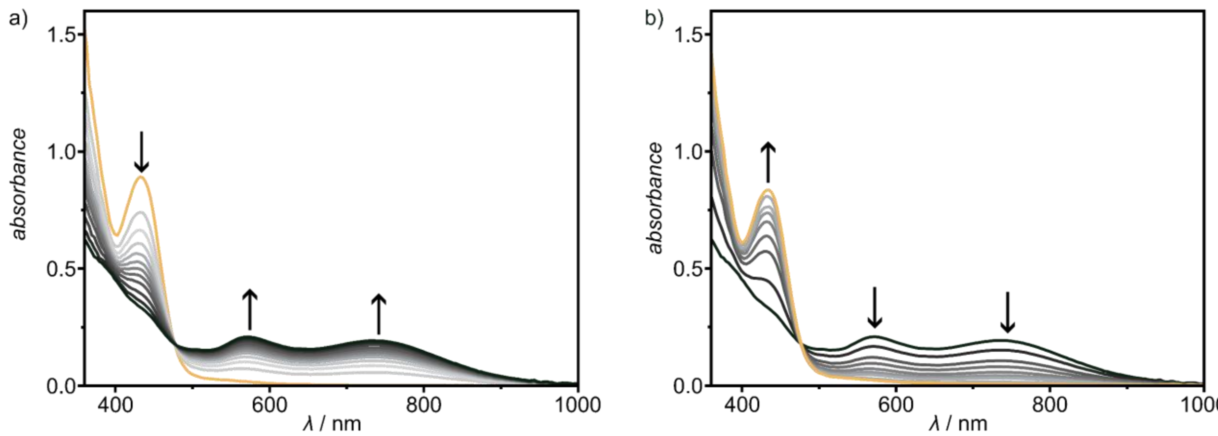

Figure S13. a) Reductive spectroelectrochemistry of **[V(dgpy)$_2$][OTf]$_3$** in CH_3CN/[*n*-Bu_4N][PF_6] (orange → black; λ_{max} = 571, 736 nm, λ_{isosb} = 478 nm) at 293 K. b) Reverse process (black → orange, λ_{max} = 435 nm, λ_{isosb} = 478 nm) at 293 K indicates reversibility of the **[V(dgpy)$_2$]$^{3+/2+}$** redox process.

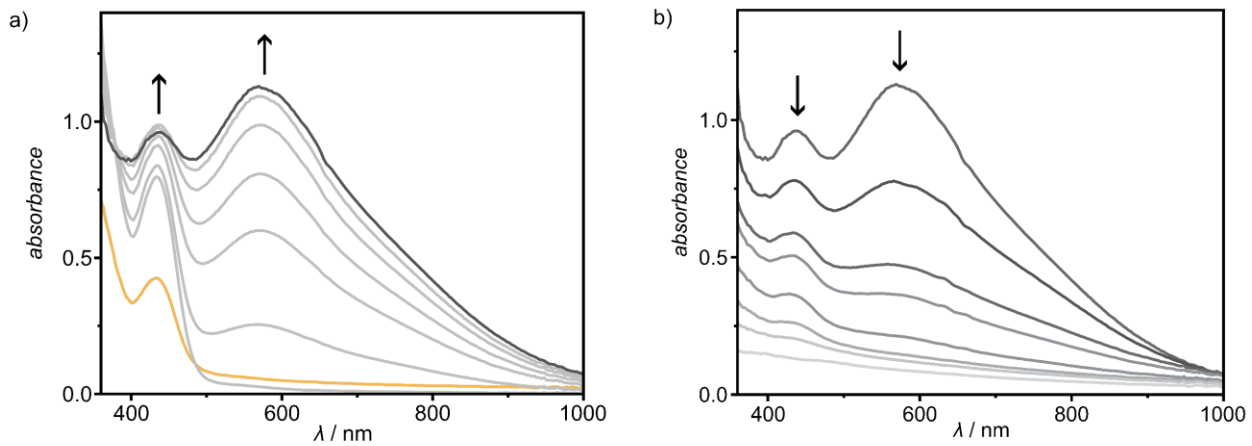

Figure S14. a) Oxidative spectroelectrochemistry of **[V(dgpy)$_2$][OTf]$_3$** in CH_3CN/[*n*-Bu_4N][PF_6] (orange → black; λ_{max} = 439, 569 nm) at 293 K. b) Reverse process (dark grey → light grey) at 293 K indicates irreversibility of the **[V(dgpy)$_2$]$^{4+/3+}$** redox process under these conditions.

Detailed discission of the gas phase experiments

Electron transfer dissociation (ETD) mass spectra were obtained from the **$[V(dgpy)_2]^{3+}$** parent ion and the **$\{[V(dgpy)_2][OTf]\}^{2+}$** ion cluster (Figure S15, Table S2). The former is cleanly reduced to **$[V(dgpy)_2]^{2+}$** (m/z = 379) and even further to the monocation **$[V(dgpy)_2]^{+}$** (m/z = 757), while the latter is singly reduced to **$\{[V(dgpy)_2][OTf]\}^{+}$** (m/z = 906).

Collision-induced dissociation (CID) experiments of dgpy vanadium ions show a stability comparable to dgpy chromium(III) species (Figure S16). The dgpy ligand of the **$[V(dgpy)_2]^{3+}$** trication undergoes fragmentation, while this fragmentation channel is not observed for the dication **$[V(dgpy)_2]^{2+}$** (Figure 3a,3d, Figure S16). Upon increasing the CID amplitude, the total ion signal is diminished indicative of a fragment ion below our detection threshold of $m/z < 50$ for **$[V(dgpy)_2]^{2+}$**. A complete assignment of the observed fragments is given in Table S3. In contrast to the chromium and manganese complexes with hexafluorophosphate counter ions showing dissociation into PF_5 and F^- followed by fluoride coordination, such counter ion fragmentation and substitution reactions play only minor roles for the vanadium complexes with triflate counter ions. Yet, CID mass spectrometric experiments confirm the higher dgpy ligand lability of reduced species and ion pairs with the counter ion especially for monocationic species and clusters (Figure S16). The major fragmentation channels for **$\{[V(dgpy)_2][OTf]\}^{+}$** and **$\{[V(dgpy)_2][OTf]_2\}^{+}$** indeed involve loss of an intact dgpy ligand giving **$\{[V(dgpy)][OTf]\}^{+}$** (m/z = 553, Figure 3e) and **$\{[V(dgpy)][OTf]_2\}^{+}$** (m/z = 702, Figure 3c) suggesting that the triflate counter ions in the ion clusters can substitute the dgpy ligand in the gas phase. The major fragmentation pattern of **$\{[V(dgpy)_2][OTf]\}^{2+}$** involves dissociation of an intact triflate anion giving **$[V(dgpy)_2]^{3+}$** (m/z = 252, Figure 3b). As minor channel, dgpy dissociation combined with electron capture (formally $dgpy^{\bullet+}$ loss) is also observed giving **$\{[V(dgpy)][OTf]\}^{+}$** (m/z = 553, Figure 3e). Overall, the fragmentation pattern of the dication **$\{[V(dgpy)_2][OTf]\}^{2+}$** is much more complex compared to the singly charged ion clusters.

The relative UV/vis photostability of the ion clusters **$\{[V(dgpy)_2][OTf]\}^{2+}$**, **$\{[V(dgpy)_2][OTf]_2\}^{+}$** and **$\{[V(dgpy)_2][OTf]\}^{+}$** in the gas phase was investigated using ultraviolet photodissociation (UVPD) mass spectrometry in the 220 – 400 nm spectral region (Figure S17). Compared to the UVPD spectra of the dgpy manganese(II,III,IV) complexes,[55] the overall signal intensity was relatively weak, pointing to a higher UV photostability of the present vanadium complexes. Furthermore, the fragmentation intensity is about an order of magnitude lower for the doubly charged ion cluster **$\{[V(dgpy)_2][OTf]\}^{2+}$** than for the singly charged ion clusters. The naked complex ions **$[V(dgpy)_2]^{3+}$** and **$[V(dgpy)_2]^{2+}$** did not yield UVPD spectra at all which agrees with their higher stability in the CID experiments. The main UV absorptions below 300 nm in solution are reproduced by the UVPD spectra (Figure S17), suggesting that high energy UV photons can lead to fragmentation. The cluster ions are photostable upon irradiation into lower energy absorption bands above 300 nm for the vanadium(III) cluster ions **$\{[V(dgpy)_2][OTf]\}^{2+}$** and **$\{[V(dgpy)_2][OTf]_2\}^{+}$** and above 330 nm for the vanadium(II) ion cluster **$\{[V(dgpy)_2][OTf]\}^{+}$**. Again, this attests high UV photostability of the cluster ions in the gas phase.

Even with high energy excitation at 265 nm, laser power dependent fragmentation studies revealed for most fragmentation pathways that more than one UV photon is required to induce fragmentation (multiphoton process) (Figure S18). Dissociation of a bidentate bpy ligand from $[Ru(bpy)_3]^{2+}$ has been suggested to require two photons and a similar situation might be encountered here for the loss of the chelate ligand dgpy from metal centers. The monocationic ion clusters **$\{[V(dgpy)_2][OTf]\}^{+}$** and **$\{[V(dgpy)_2][OTf]_2\}^{+}$** display identical CID/UVPD fragmentation patterns, while the CID/UVPD patterns differ for the dicationic

vanadium(III) cluster ion **{[V(dgpy)$_2$][OTf]}$^{2+}$** (Figure 3b,3c,3e). The dicationic ion cluster **{[V(dgpy)$_2$][OTf]}$^{2+}$** dissociates a triflate anion giving **[V(dgpy)$_2$]$^{3+}$** (*m*/*z* 252) under CID conditions, but yields **[V(dgpy)$_2$]$^{2+}$** (*m*/*z* 379) under UVPD conditions as the major fragmentation channel. Clearly the UVPD process is associated with a charge transfer from OTf$^-$ to the vanadium(III) complex dissociating a neutral trifluoromethanesulfonyl radical OTf$^•$ and leaving the vanadium(II) complex. In contrast to the multiphotonic processes of the other UV light-induced dissociation channels, this charge-transfer dissociation of the ion-pair is induced by a single 265 nm photon (Figure S18). The electron affinity of the free OTf$^•$ radical is estimated to be about 5 eV corresponding to ca. 250 nm photons. This estimation renders the charge-transfer from OTf$^-$ to the vanadium(III) complex conceivable at the investigated photon energies. These gas phase studies using high energy UV/vis photons highlight the overall photostability of the vanadium(III/II) complexes and the ease of the interconversion between redox states (Figure 2).

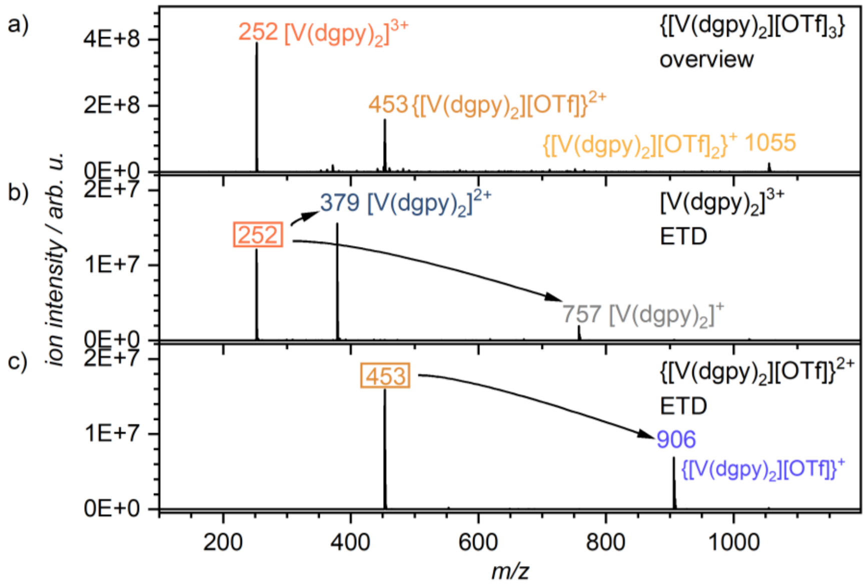

Figure S15. a) Overview mass spectrum of a solution of **[V(dgpy)$_2$][OTf]$_3$** in CH_3CN. Resulting spectra of the ETD processes of b) **[V(dgpy)$_2$]$^{3+}$** and c) **{[V(dgpy)$_2$][OTf]}$^{2+}$**. The arrows indicate reduction steps.

Table S2. Mass-to-charge ratios of the observed vanadium species in the ESI$^+$ mass spectra including the ones formed by *in situ* by electron transfer.

assignment	*m*/*z*
[V(dgpy)$_2$]$^{3+}$	252
{[V(dgpy)$_2$][OTf]}$^{2+}$	453
{[V(dgpy)$_2$][OTf]$_2$}$^+$	1055
[V(dgpy)$_2$]$^{2+}$	379
{[V(dgpy)$_2$][OTf]}$^+$	906
[V(dgpy)$_2$]$^+$	757

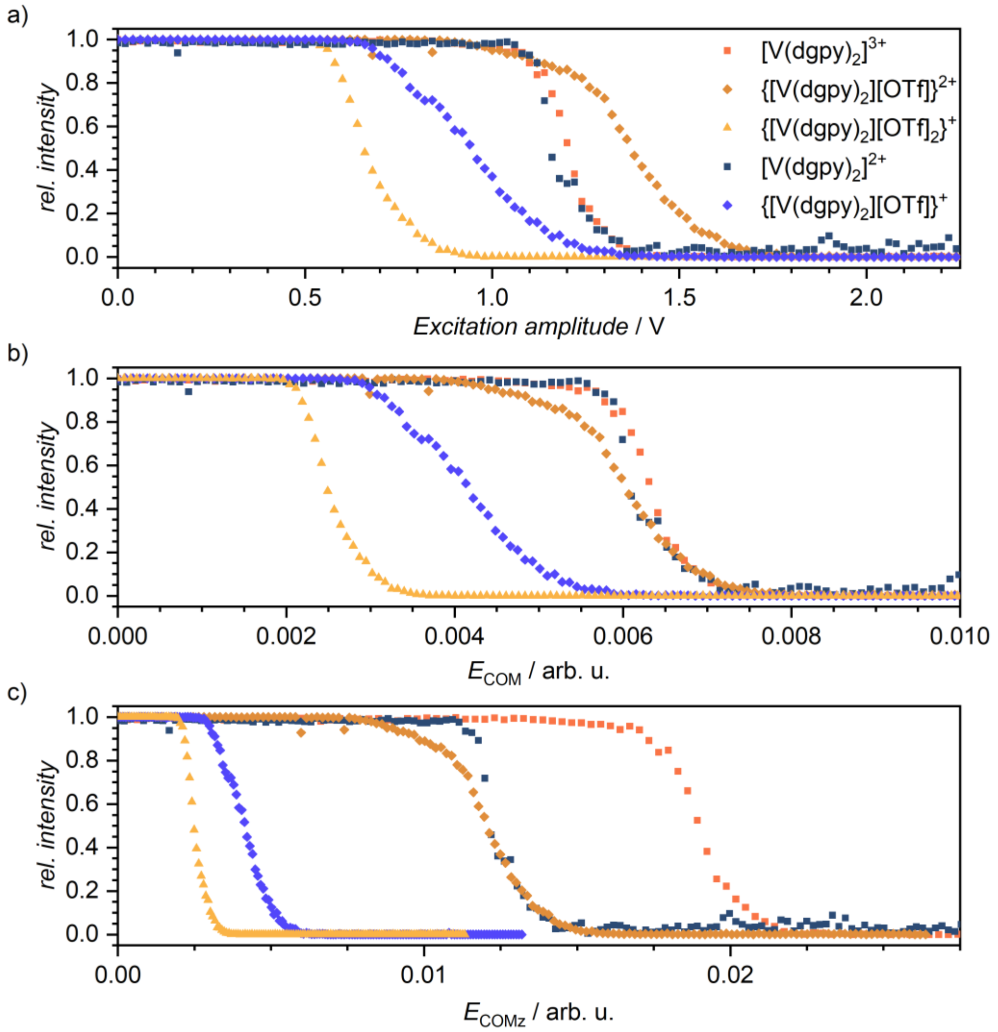

Figure S16. CID breakdown curves for the five investigated vanadium species with different calculated energy axes. a) the RF amplitude as given by the software/instrument, b) center-of-mass corrected E_{COM} values to account for the different masses of the complexes and c) mass and charge corrected values E_{COMz} to account for the different charge states of the investigated ions. Only the decrease of the parent ion (breakdown) is plotted against the different respective axis (see method section).

Table S3. Major fragmentation channels from CID experiments for the five different investigated vanadium species. The second column gives the *m*/*z* ratio of the respective fragment followed by an assignment of a sum formula. The last column gives a classification of the type of fragmentation: (i) Loss of an intact dgpy ligand, (ii) fragmentation of the dgpy ligand, (iii) intact loss of OTf^{-} (counter ion), (iv) fragmentation of OTf^{-} and (v) fragmentation involving charge transfer. The main fragmentation channel is indicated in bold. Note that in the case of **$[V(dgpy)_2]^{2+}$** we observe a near complete loss of intensity upon CID. This hints at a fragmentation channel giving an ionic fragment below the detection limit of the instruments, i.e. smaller than *m*/*z* 50. Thus, we postulate V^{2+} as a likely product of this channel.

	fragment *m*/*z*	assignment	
**$[V(dgpy)_2]^{3+}$**	**330**	**$\{[V(dgpy)_2(\text{-guanidyl})](CH_3CN)\}^{2+}$**	**fragmentation dgpy**
m/*z* 252	354	$(dgpyH)^{+}$	loss of intact dgpy
	392	(dgpyK)+	
	453	$\{?\}^{2+}$	
**$[V(dgpy)_2]^{2+}$**	252	$[V(dgpy)_2]^{3+}$	charge transfer
m/*z* 379	309	$\{[V(dgpy)_2(\text{-guanidyl})]\}^{2+}$	fragmentation of dgpy
	**26**	**V^{2+}**	**loss of intact dgpy**<br>**dark → below detection limit**
**$\{[V(dgpy)_2][OTf]\}^{+}$**<br>*m*/*z* 906	420	$\{\{[V(dgpy)]O\}^{+}$	loss of intact dgpy<br>fragmentation of OTf
	435	$\{[V(dgpy)](O,F)\}^{+}$	loss of intact dgpy<br>fragmentation of OTf
	439	$\{[V(dgpy)](O,F)\}^{+}$	loss of intact dgpy<br>fragmentation of OTf
	469	$\{?\}^{+}$	
	**553**	**$\{[V(dgpy)][OTf]\}^{+}$**	**loss of intact dgpy**
**$\{[V(dgpy)_2][OTf]_2\}^{+}$**<br>*m*/*z* 1055	439	$\{[V(dgpy)](O,F)\}^{+}$	loss of intact dgpy<br>loss of intact OTf^{-} and fragmentation of OTf
	569	$\{[V(dgpy)][OTf](O,F)\}^{+}$	loss of intact dgpy and fragmentation of OTf
	**702**	**$\{[V(dgpy)][OTf]_2\}^{+}$**	**loss of intact dgpy**
**$\{[V(dgpy)_2][OTf]\}^{2+}$**	252	$[V(dgpy)_2]^{3+}$	loss of intact OTf^{-}
m/*z* 453	309	$\{[V(dgpy)_2(\text{-guanidyl})](OTf)\}^{2+}$	fragmentation of dgpy
	**354**	**$(dgpyH)^{+}$**	**loss of intact dgpy**
	379	$[V(dgpy)_2]^{2+}$	loss of intact OTf^{-}<br>charge transfer
	553	$\{[V(dgpy)][OTf]\}^{+}$	loss of intact dgpy

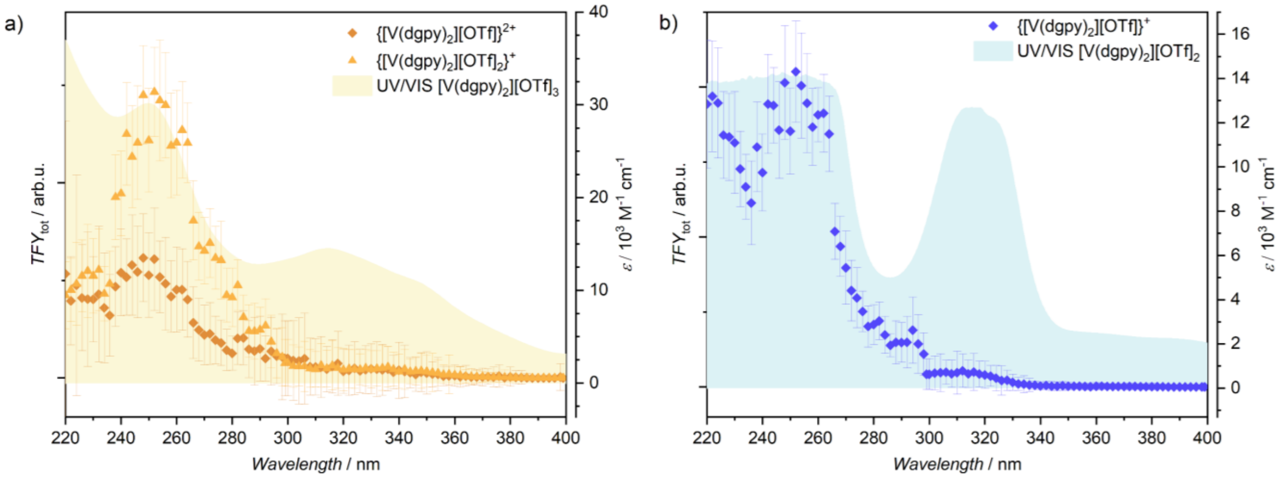

Figure S17. a) UVPD spectra of **{[V(dgpy)$_2$][OTf]}$^{2+}$** (orange diamonds), **{[V(dgpy)$_2$][OTf]$_2$}$^+$** (light orange triangles) from 220 to 400 nm and b) of **{[V(dgpy)$_2$][OTf]}$^+$** (blue diamonds). The condensed phase UV/vis spectra of **[V(dgpy)$_2$][OTf]$_3$** (Figure 4a) and **[V(dgpy)$_2$][OTf]$_2$** (Figure 4b) in CH_3CN in the same absorption range are shown for comparison (colored areas in a) orange and b) blue). The intensity of the UV/vis spectrum in panel b) below 270 nm has reached the detector limit and is not comparable.

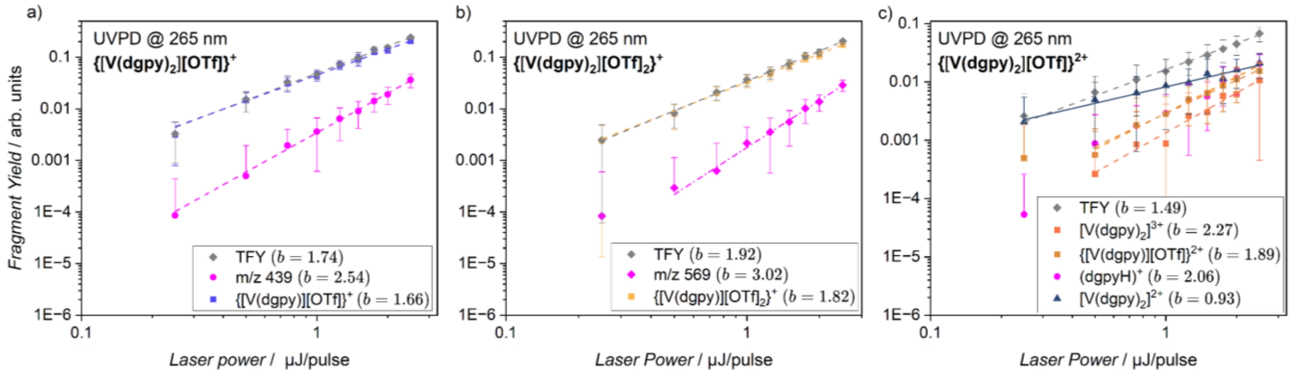

Figure S18. Power dependent measurements of the three vanadium species with triflate ions for which UVPD spectra could be measured. a) **{[V(dgpy)$_2$][OTf]}$^+$**, b) **{[V(dgpy)$_2$][OTf]$_2$}$^+$** and c) **{[V(dgpy)$_2$][OTf]}$^{2+}$**. Experimental data points are given by the symbols and the allometric fits $y = a \cdot x^b$ are given as dashed lines. In each panel, the total fragment yield (TFY) is given as grey diamonds. In all cases, the allometric fit gives exponents b larger than 1.5 indicating that multiple photons are needed for dissociation, which agrees with CID and UVPD giving very similar fragmentation patterns. The notable exception is the fragmentation channel associated to charge transfer in **{[V(dgpy)$_2$][OTf]}$^{2+}$** giving **[V(dpgy)$_2$]$^{2+}$** (*m*/*z* 378) (green solid line in panel c)) for which the allometric fit gives $b = 0.93$. Note that this channel is only of minor importance in CID.

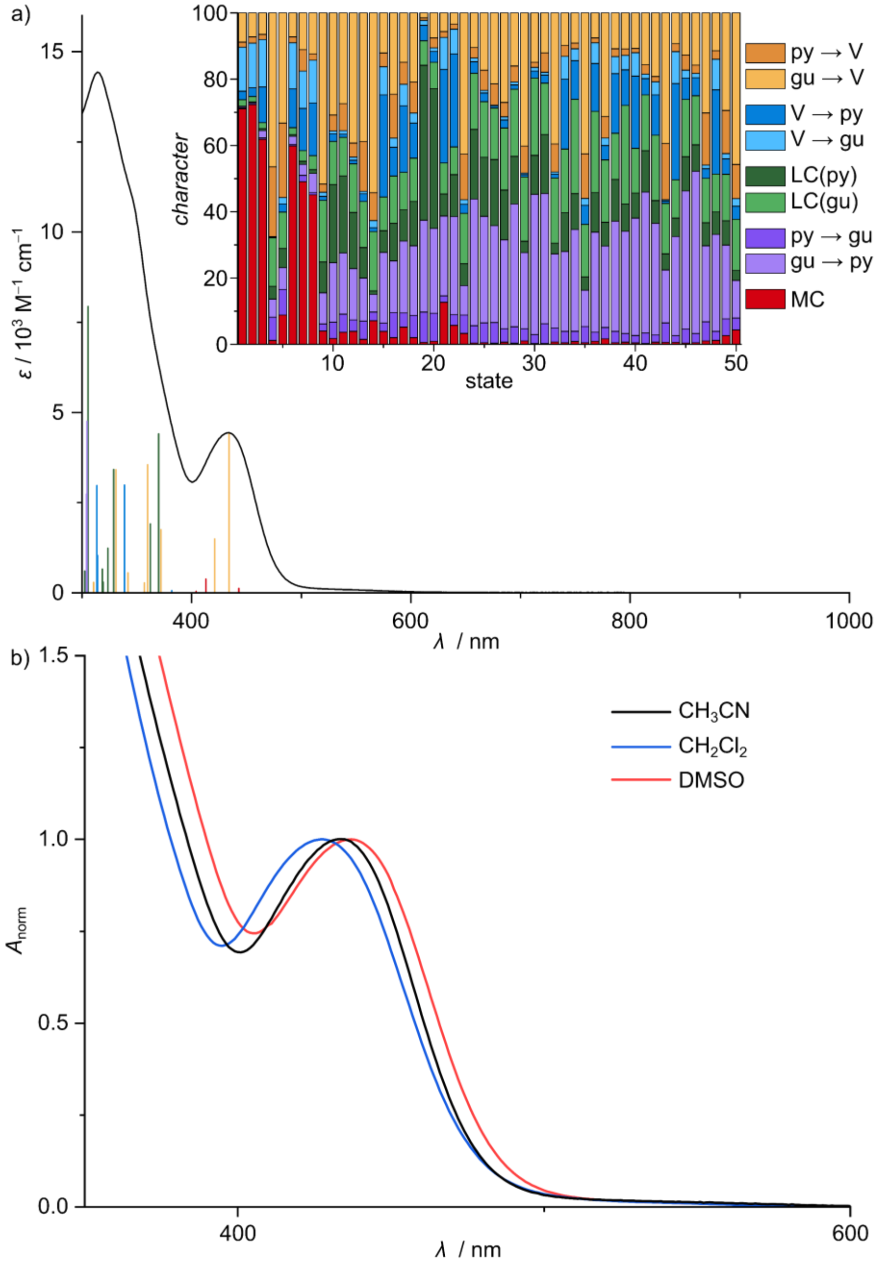

Figure S19. a) UV/vis/NIR absorption spectrum of **[V(dgpy)$_2$][OTf]$_3$** in CH_3CN at 293 K (black). TD-DFT calculated oscillator strengths (vertical bars, color-coded according to a charge-transfer number analysis; MC = red, LC = dark/light green (py/guanidine), MLCT = dark/light blue (V→py/guanidine), LMCT = dark/light orange (py/guanidine→V), ILCT = purple (guanidine→py)). Calculated transitions are shifted to higher energies by 883 cm^{-1} to better match the experimental data. b) UV/vis absorption spectra of **[V(dgpy)$_2$][OTf]$_3$** normalized to the lowest-energy maximum in CH_3CN (black), CH_2Cl_2 (blue) and DMSO (red) at 293 K in the CT band region with maxima at 434, 428 and 437 nm.

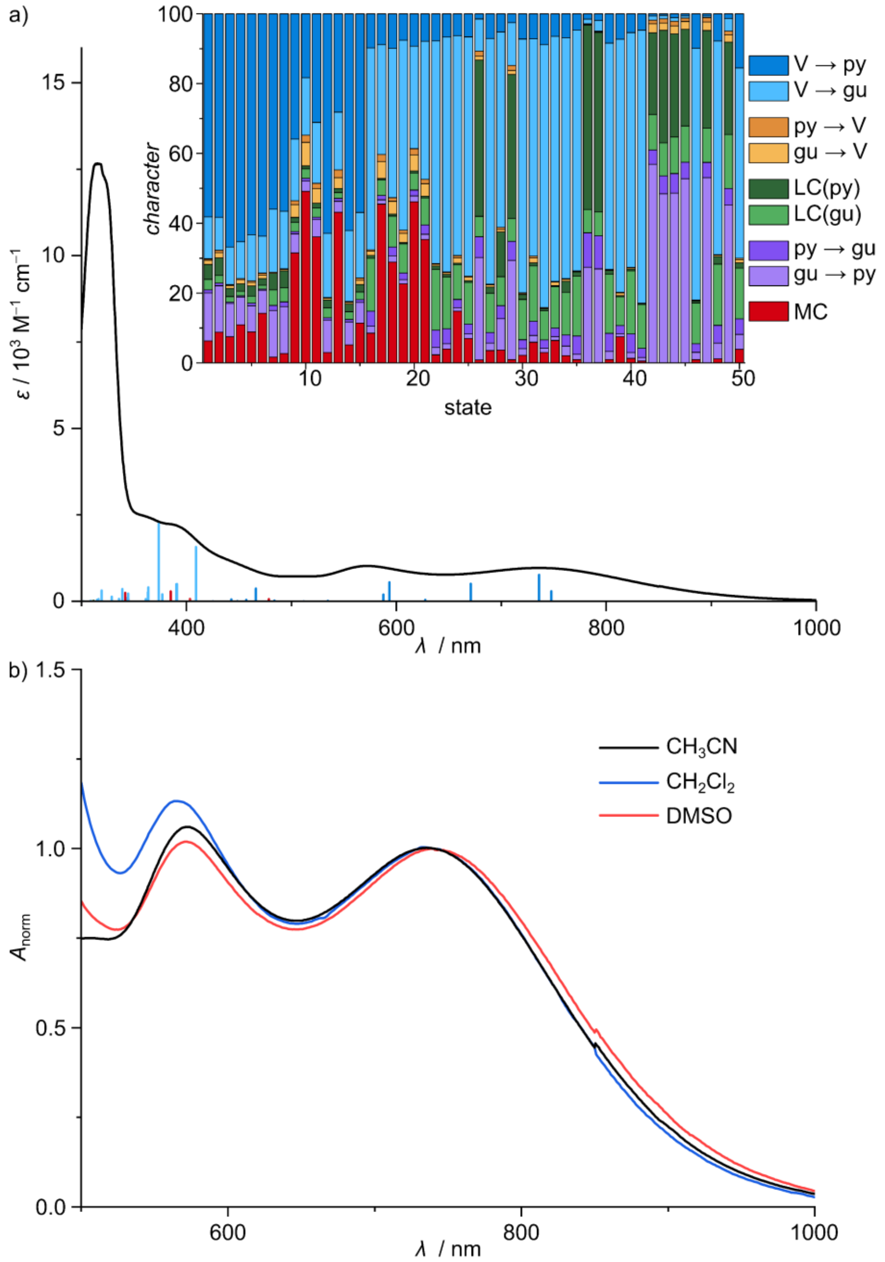

Figure S20. a) UV/vis/NIR absorption spectrum of **[V(dgpy)$_2$][OTf]$_2$** in CH_3CN at 293 K (black). TD-DFT calculated oscillator strengths (vertical bars, color-coded according to a charge-transfer number analysis; MC = red, LC = dark/light green (py/guanidine), MLCT = dark/light blue (V→py/guanidine), LMCT = dark/light orange (py/guanidine→V), ILCT = purple (guanidine→py)) Calculated transitions are shifted to higher energies by 1023 cm^{-1} to better match the experimental data. b) UV/vis absorption spectra of **[V(dgpy)$_2$][OTf]$_2$** normalized to the lowest-energy maximum in CH_3CN (black), CH_2Cl_2 (blue) and DMSO (red) at 293 K in the CT band region with maxima at 573/736, 565/734 and 571/740 nm.

Table S4. TD-DFT calculated electronic transitions of **[V(dgpy)$_2$]$^{3+}$**. Hydrogen atoms are omitted. Electron density gain = orange, electron density loss = purple; isosurface value 0.003 a.u..

**TD-DFT difference density**	**λ / nm** (unshifted)	**λ / nm** (shifted by 883 cm^{-1} to higher energy)	**oscillator strength f**	**number**
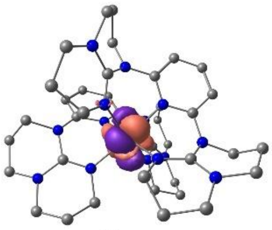	1993.1		0.00101784	1
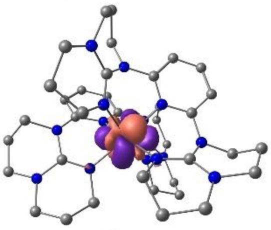	1677.2		0.00032264	2
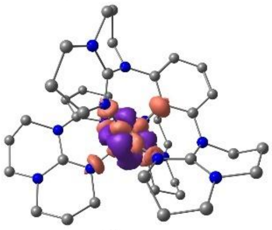	461	443.0	0.00092768	3
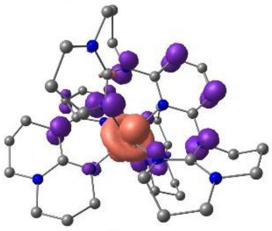	451.3	434.0	0.03297335	4
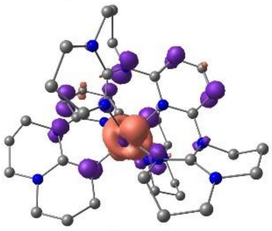	437.2	420.9	0.01121957	5
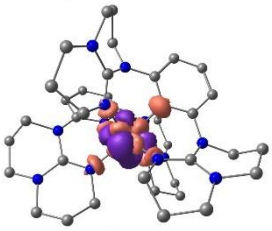	428.7	413.1	0.00284443	6
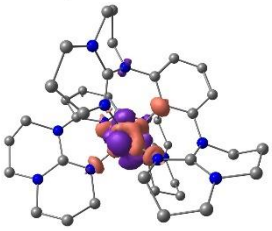	418.9	404.0	0.00033633	7

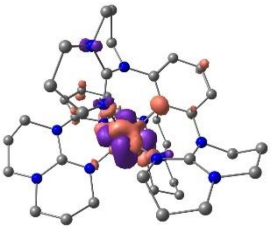	395.1	381.8	0.00044586	8
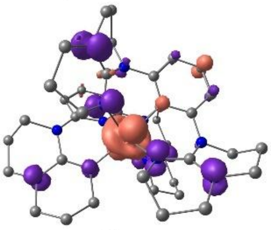	384.7	372.1	0.01314989	9
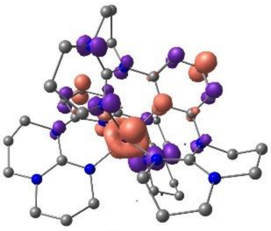	382.4	369.9	0.03304521	10
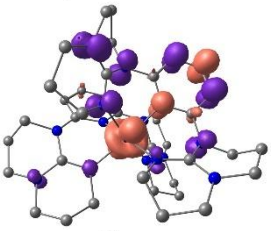	374.3	362.3	0.01435659	11
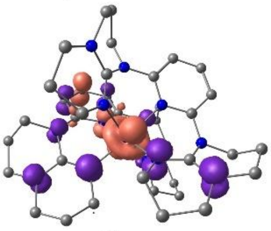	371.6	359.8	0.02662609	12
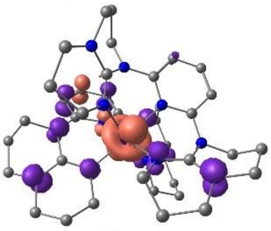	368.5	356.9	0.00210119	13
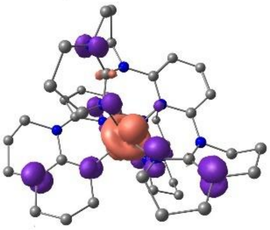	352.5	341.9	0.00417343	14
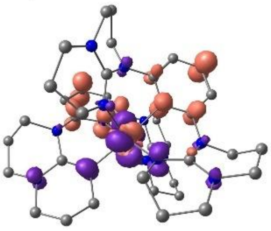	349.2	338.8	0.02243981	15

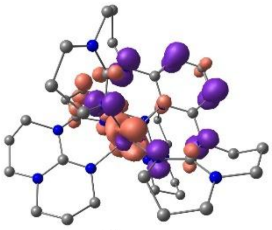	340.8	330.8	0.02557375	16
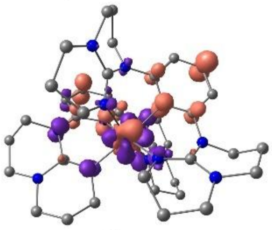	338.7	328.9	0.02564377	17
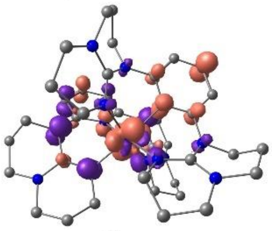	333.2	323.7	0.0093043	18
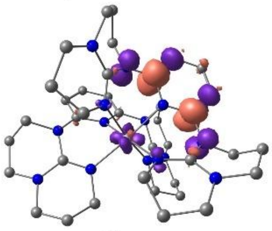	328.5	319.2	0.00221845	19
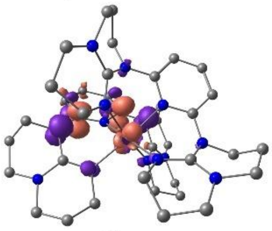	327.8	318.6	0.00495576	20
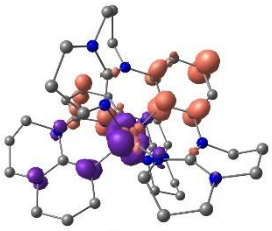	323.1	314.1	0.00775904	21
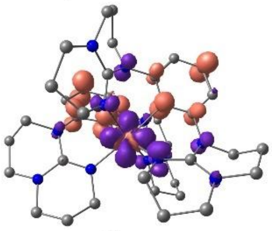	322.5	313.6	0.02229957	22
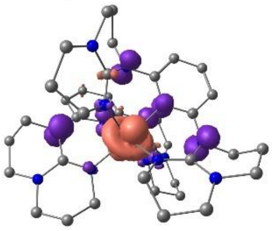	319.2	310.4	0.00220369	23

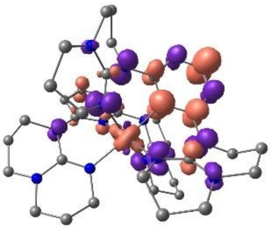	314	305.5	0.05951438	24
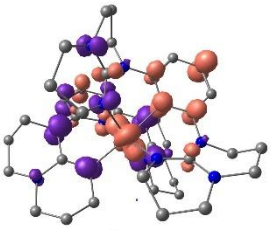	313.1	304.7	0.03563877	25
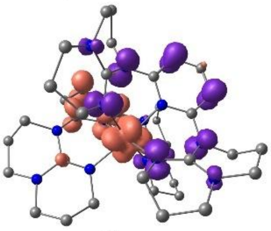	312.5	304.1	0.0205499	26
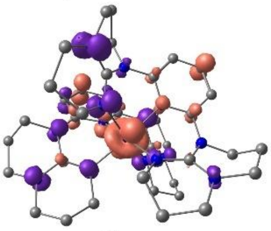	310.8	302.5	0.00455374	27
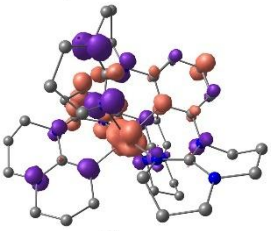	307.4	299.3	0.01849627	28
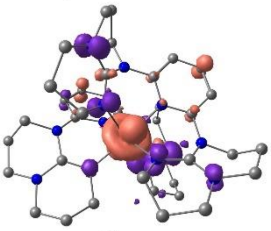	304.7	296.7	0.05583009	29
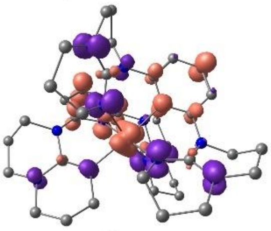	303.6	295.7	0.00595308	30
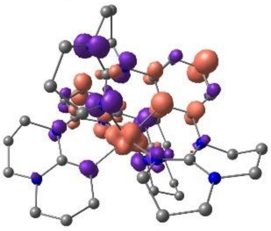	302.7	294.8	0.00681667	31

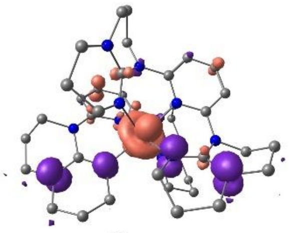	299.8	292.1	0.03144462	32
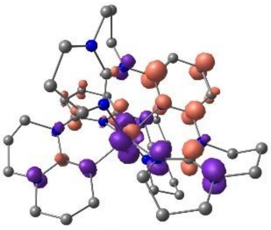	296.7	289.1	0.01965826	33
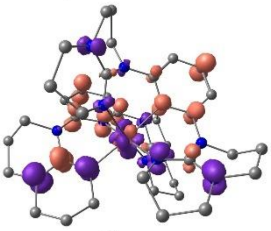	296.1	288.6	0.02212643	34
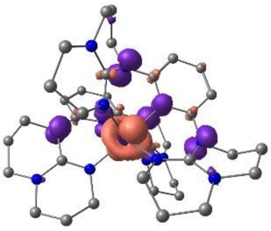	294.5	287.0	0.01987085	35
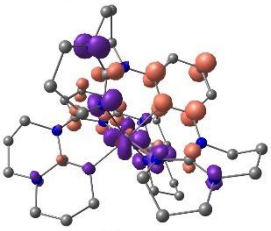	290.3	283.0	0.00032841	36
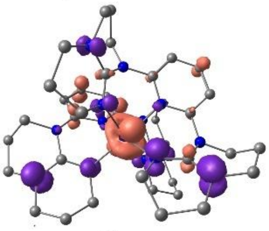	288.5	281.3	0.0177911	37
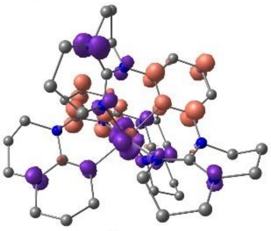	287.2	280.1	0.09935573	38
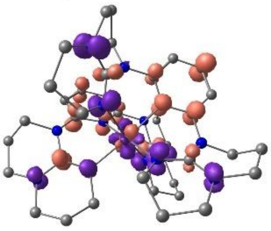	286.5	279.4	0.01397928	39

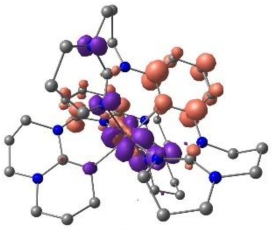	283.4	276.5	0.01521285	40
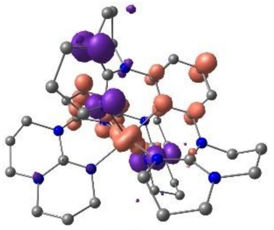	281.9	275.1	0.00437348	41
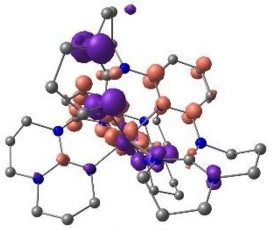	279.5	272.8	0.0061763	42
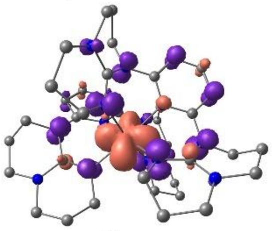	277.5	270.9	0.01218297	43
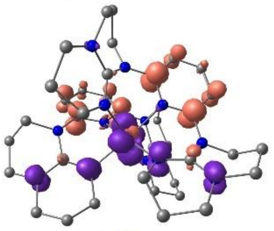	275.3	268.8	0.00031047	44
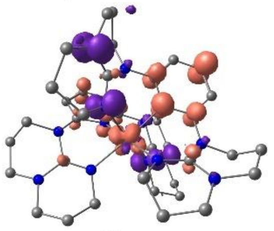	274.5	268.0	0.05514245	45
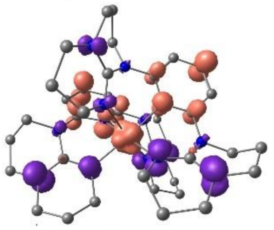	273.5	267.0	0.04951473	46
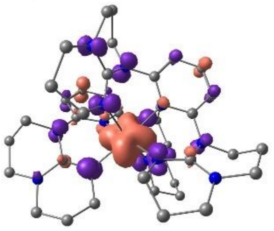	272.5	266.1	0.0030466	47

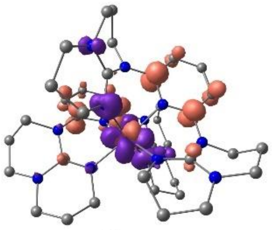	271.7	265.3	0.02793212	48
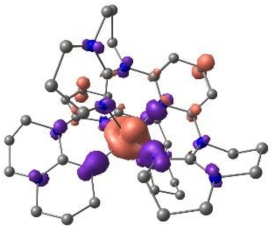	270.8	264.5	0.00074953	49
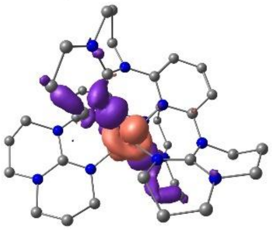	270.4	264.1	0.01789604	50

Table S5. TD-DFT calculated electronic transitions of **$[V(dgpy)_2]^{2+}$**. Hydrogen atoms are omitted. Electron density gain = orange, electron density loss = purple; isosurface value 0.003 a.u..

**TD-DFT difference density**	**λ / nm** (unshifted)	**λ / nm** (shifted by 1023 cm^{-1} to higher energy)	**oscillator strength *f***	**number**
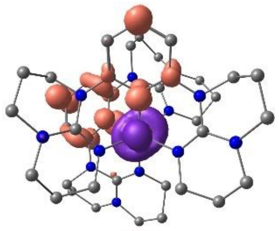	826	747.6	0.006056	1
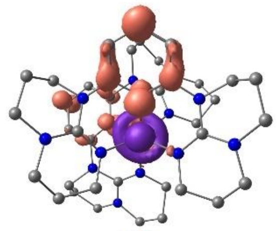	811.9	736.0	0.015944	2
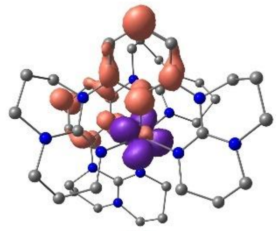	733.4	670.9	0.010606	3
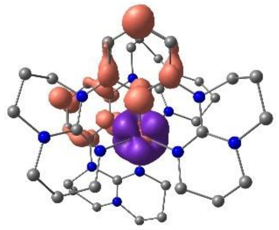	681.8	627.5	0.000883	4
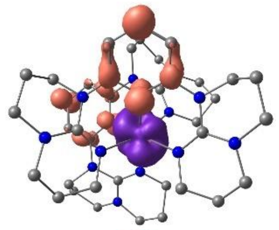	641.7	593.3	0.011488	5
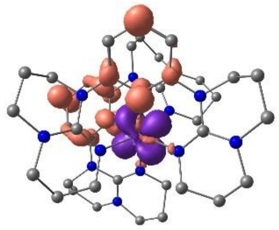	635	587.6	0.004121	6
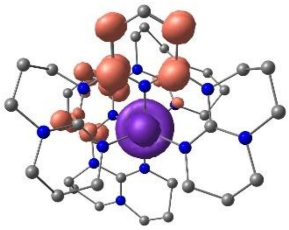	573.6	534.6	0.000314	7

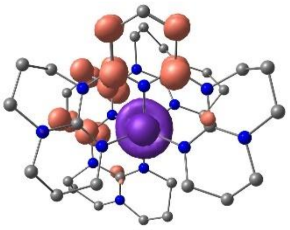	547.5	511.9	3.58E−05	8
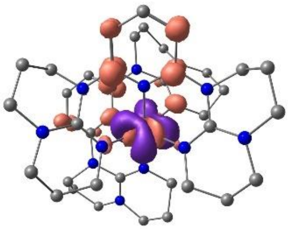	515.5	483.8	0.000374	9
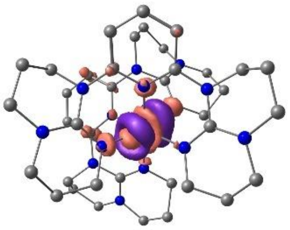	509.6	478.6	0.001313	10
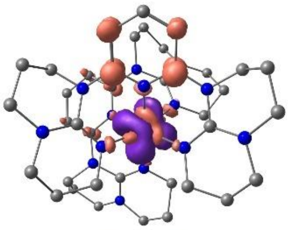	495.5	466.2	0.007693	11
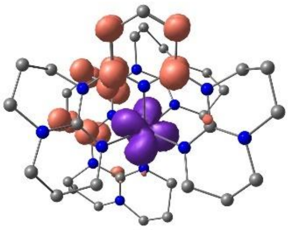	485.2	457.0	0.000854	12
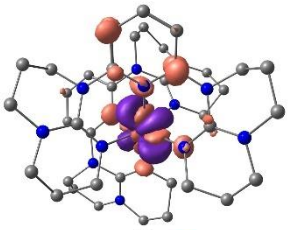	475.6	448.5	0.000153	13
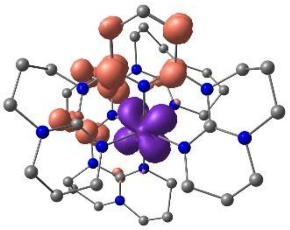	469.1	442.7	0.001227	14
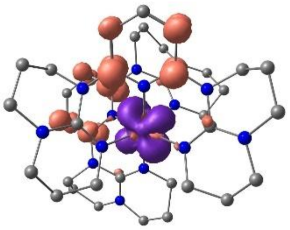	449.3	425.0	5.85E−05	15

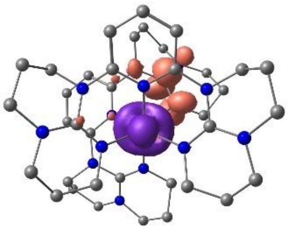	431.5	409.1	0.032921	16
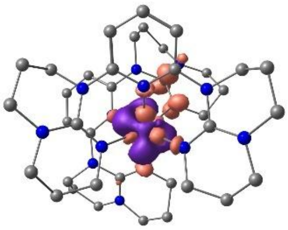	425.1	403.3	0.001461	17
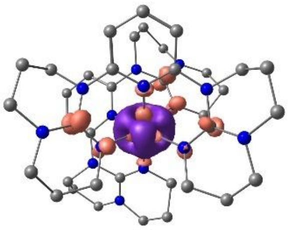	411.4	391.0	0.01054	18
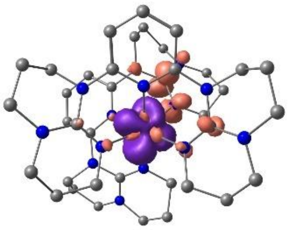	410.7	390.3	0.010403	19
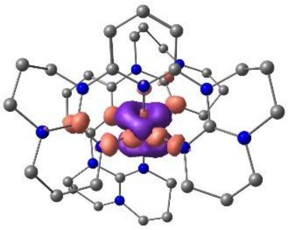	404.8	385.0	0.005953	20
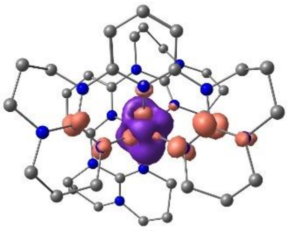	396	377.0	0.004252	21
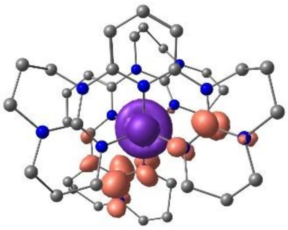	392.3	373.7	0.046907	22
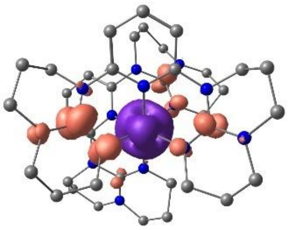	381.4	363.8	0.008488	23

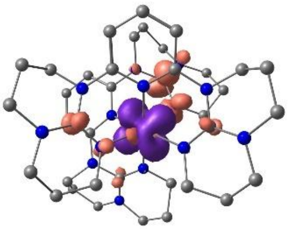	380.9	363.3	0.00536	24
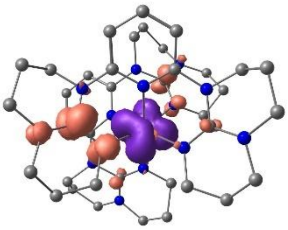	378.6	361.2	0.001281	25
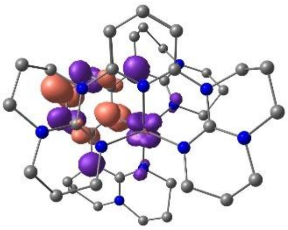	360.6	344.8	0.001484	26
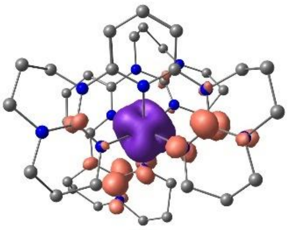	360.3	344.5	0.004616	27
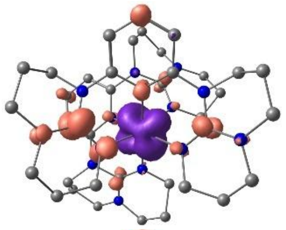	358.5	342.9	0.001846	28
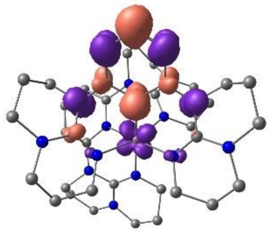	357.1	341.6	0.005027	29
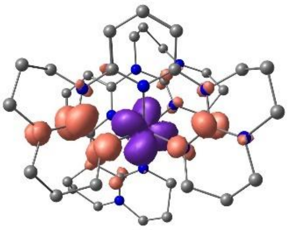	354.2	339.0	0.007443	30
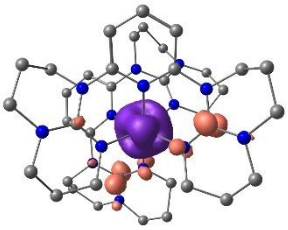	350.7	335.7	0.001444	31

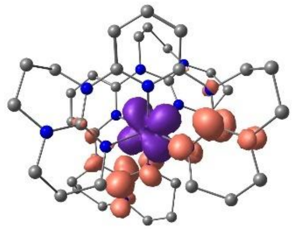	343.3	329.0	0.002696	32
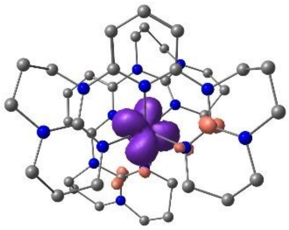	332.7	319.2	0.006518	33
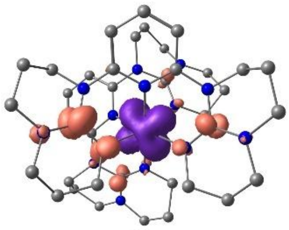	329.6	316.4	0.00137	34
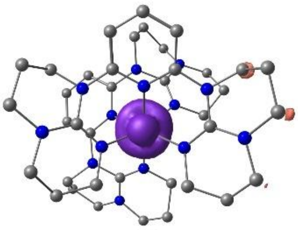	328	314.9	0.000939	35
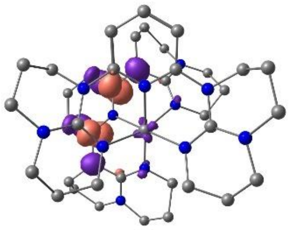	324.5	311.7	0.000247	36
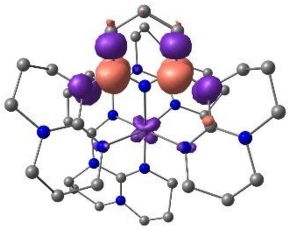	323.6	310.8	0.000173	37
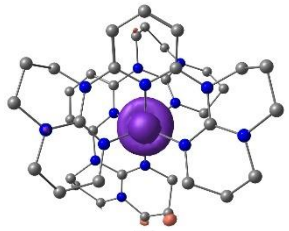	321.3	308.7	0.000345	38
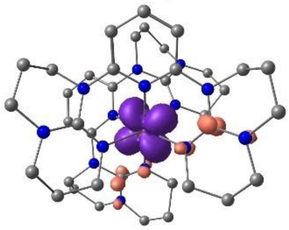	314.2	302.1	4.65E−05	39

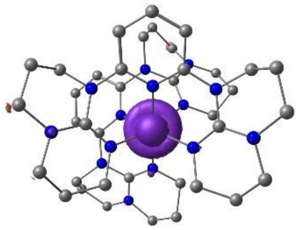	309.6	297.9	0.001149	40
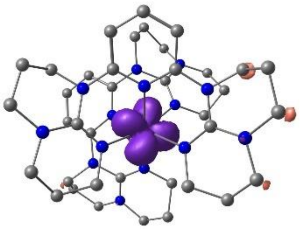	306.6	295.1	0.000578	41
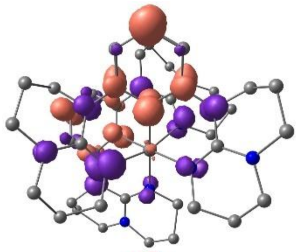	306.2	294.7	0.107495	42
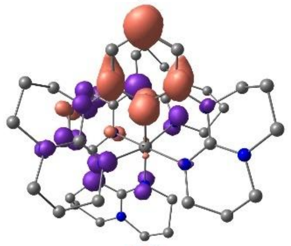	302.6	291.4	0.014507	43
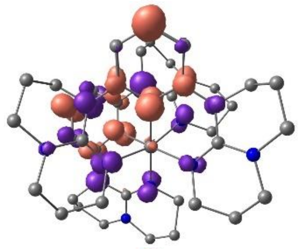	301.7	290.6	0.084544	44
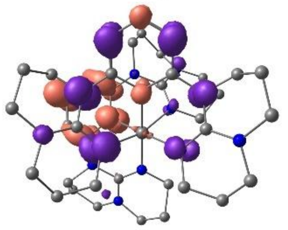	300.7	289.6	0.006895	45
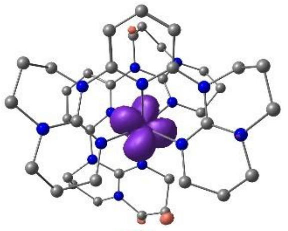	300.6	289.5	0.002408	46
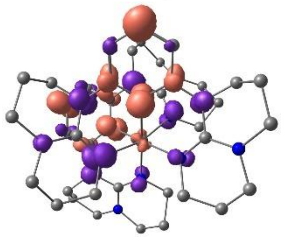	297	286.2	0.002451	47

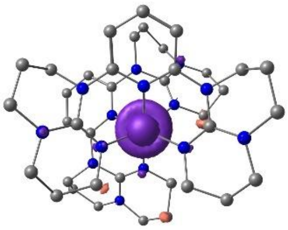	293.7	283.1	0.005763	48
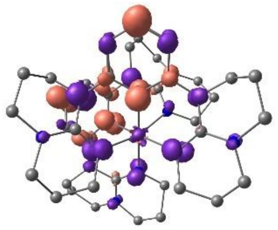	293.2	282.7	0.051849	49
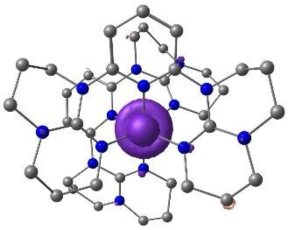	291.8	281.4	0.000532	50

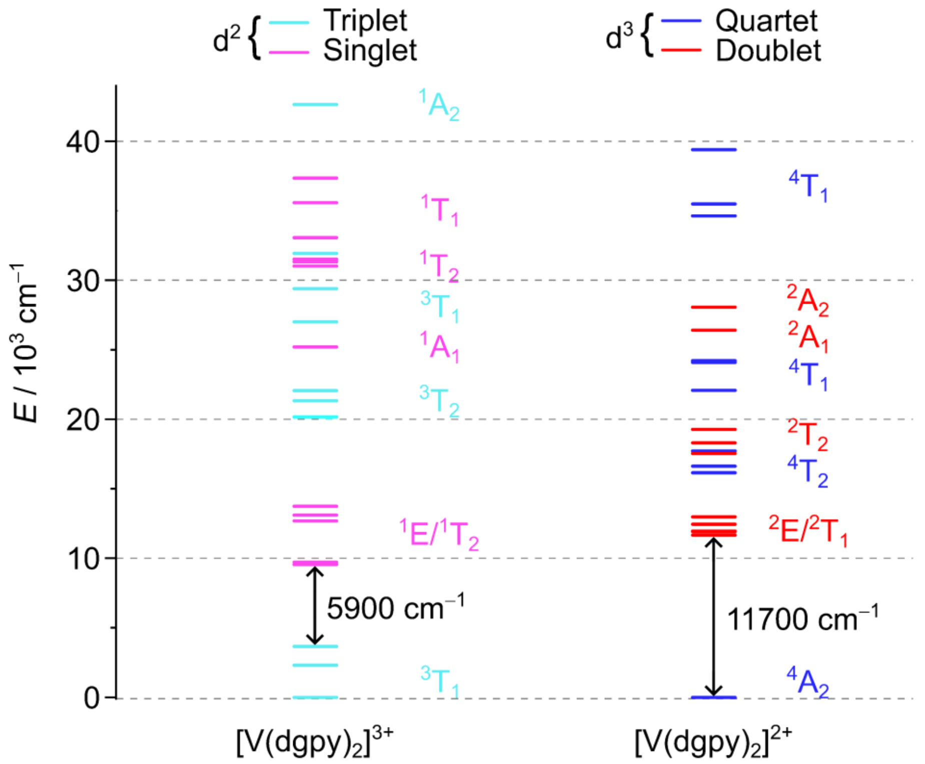

Figure S21. Energy diagrams of ligand field states derived from CASSCF(6,12)-NEVPT2 calculations at the DFT-optimized ground state geometry of ***cisfac*-[V(dgpy)$_2$]$^{3+}$** with the smallest energy gap between the lowest-energy excited state and the split ground state given in cm^{-1} (left) and derived from CASSCF(7,12)-NEVPT2 calculations at the DFT-optimized ground state geometry of ***cisfac*-[V(dgpy)$_2$]$^{2+}$** with the smallest energy gap between the lowest-energy excited state and the ground state given in cm^{-1} (right). Note that charge transfer contributions or charge transfer states are not covered by these calculations.

Table S6. Energies (in Hartree) of the 12 orbitals used in the active space of the CASSCF(6,12)-SC-NEVPT2 calculation of **$[V(dgpy)_2]^{3+}$**, orbitals depicted at a contour value of 0.03 a.u.. Hydrogen atoms are omitted.

#	*E* / H	orbital	#	*E* / H	orbital
198	–0.5568	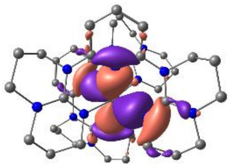	204	0.1556	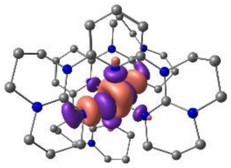
199	–0.5507	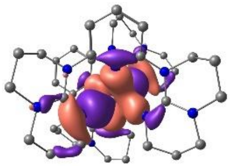	205	0.9099	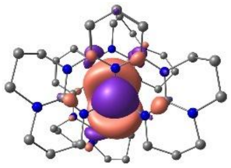
200	0.0357	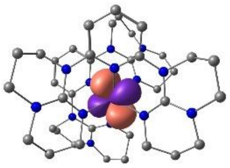	206	0.9225	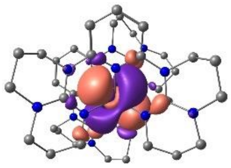
201	0.0385	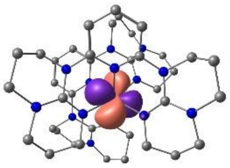	207	0.9298	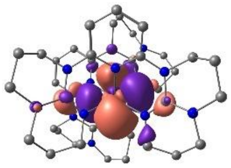
202	0.0514	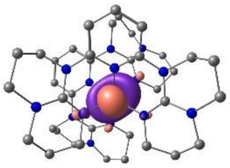	208	1.4130	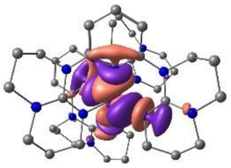
203	0.1501	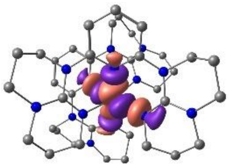	209	1.4896	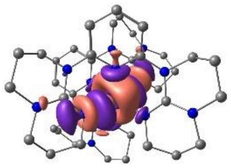

Table S7. CASSCF(6,12)-NEVPT results of **[V(dgpy)$_2$]$^{3+}$** with the main configuration of the 3d orbitals with the corresponding weight (included all configurations, so that the weight is >0.9 for each state), energies in cm^{-1} relative to the $^3T_1(1)$ ground state. Turquoise = triplet character, pink = singlet character.

**Term symbol (*O* point group)**	**main configuration of 3d orbitals (weights) (d_{xy}, d_{xz}, d_{yz}, d_{x2-y2}, d_{z2})**	***E* / cm^{-1}**
$^3T_1(1)$	11000 (0.91)	0
$^3T_1(2)$	10100 (0.96)	2308
$^3T_1(3)$	01100 (0.91)	3655
$^1E(1)$	20000 (0.58)<br>02000 (0.34)	9554
$^1T_2(1)$	11000 (0.95)	9722
$^1T_2(2)$	10100 (0.91)	12688
$^1E(2)$	02000 (0.41)<br>00200 (0.36)<br>20000 (0.16)	13113
$^1T_2(3)$	01100 (0.95)	13741
$^3T_2(1)$	01010 (0.54)<br>10001 (0.42)	20170
$^3T_2(2)$	10001 (0.52)<br>01010 (0.30)<br>00101 (0.14)	21336
$^3T_2(3)$	00110 (0.51)<br>01001 (0.32)<br>10010 (0.14)	22076
$^3T_1(1)$	10010 (0.67)<br>01001 (0.26)	25201
$^1A_1(1)$	00200 (0.53)<br>02000 (0.18)<br>20000 (0.18)	27011
$^3T_1(2)$	00110 (0.44)<br>01001 (0.36)<br>10010 (0.11)	29408
$^1T_2(1)$	01010 (0.75)<br>10010 (0.17)	31021
$^1T_2(2)$	10001 (0.52)<br>10010 (0.18)<br>01010 (0.16)	31338
$^1T_2(3)$	10010 (0.32)<br>10001 (0.26)<br>01001 (0.19)<br>00110 (0.19)	31528
$^3T_1(3)$	00101 (0.80)<br>01010 (0.12)	31942
$^1T_1(1)$	01001 (0.47)<br>10010 (0.44)	33065
$^1T_1(2)$	00110 (0.68)<br>01001 (0.25)	35583
$^1T_1(3)$	00101 (0.95)	37361
$^3A_2(1)$	10010 (0.98)	42649

Table S8. Energies of the 12 orbitals used in the active space of the CASSCF(7,12)-SC-NEVPT2 calculation of **[V(dgpy)$_2$]$^{2+}$**, orbitals depicted at a contour value of 0.03 a.u.. Hydrogen atoms are omitted.

#	*E* / H	orbital	#	*E* / H	orbital
198	–0.4983	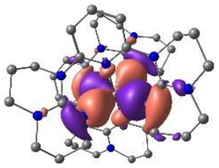	204	0.3084	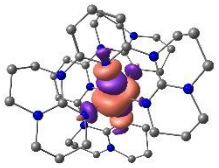
199	–0.4984	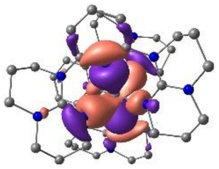	205	0.8552	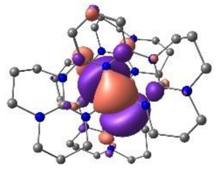
200	0.0879	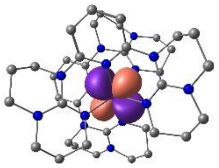	206	0.9504	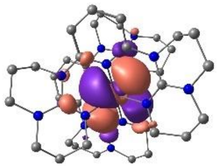
201	0.1032	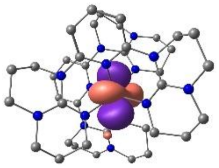	207	0.9298	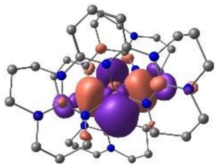
202	0.1182	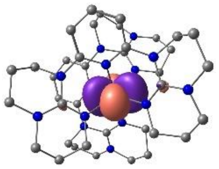	208	0.9312	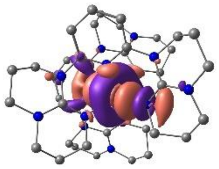
203	0.2931	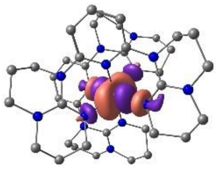	209	1.3588	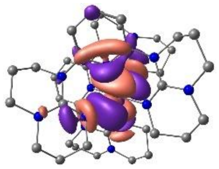

Table S9. CASSCF(7,12)-NEVPT results of **[V(dgpy)$_2$]$^{2+}$** with the main configuration of the 3d orbitals with the corresponding weight (included all configurations, so that the weight is >0.8 for each state); energies in cm^{-1} relative to the $^4A_2$ ground state. Blue = quartet character, red = doublet character.

**Term symbol (*O* point group)**	**main configuration of 3d orbitals (weights) (*d*$_{xy}$, *d*$_{yz}$, *d*$_{xz}$, *d*$_z$, *d*$_{x2-y2}$)**	***E* / cm^{-1}**
$^4A_2$	11100 (0.98)	0
$^2T_1(1)$	11100 (0.32)<br>21000 (0.20)<br>20100 (0.20)<br>02100 (0.10)	11675
$^2E(1)$	11100 (0.58)<br>12000 (0.13)<br>20100 (0.11)	11916
$^2T_1(2)$	20100 (0.31)<br>11100 (0.24)<br>21000 (0.16)<br>02100 (0.16)	11955
$^2T_1(3)$	11100 (0.24)<br>21000 (0.23)<br>10200 (0.18)<br>12000 (0.17)	12445
$^2E(2)$	11100 (0.50)<br>12000 (0.18)<br>10200 (0.17)	12991
$^4T_2(1)$	11010 (0.68)<br>10100 (0.14)	16148
$^4T_2(2)$	10101 (0.56)<br>11010 (0.19)<br>01110 (0.13)	16637
$^2T_2(1)$	02100 (0.36)<br>20100 (0.18)<br>01200 (0.11)<br>10200 (0.09)<br>12000 (0.05)<br>21000 (0.05)	17547
$^4T_2(3)$	01101 (0.40)<br>01110 (0.23)<br>10110 (0.21)	17732
$^2T_2(2)$	10200 (0.38)<br>12000 (0.30)<br>02100 (0.05)	18316
$^2T_2(3)$	01200 (0.45)<br>02100 (0.16)<br>21000 (0.15)<br>20100 (0.05)	19286
$^4T_1(1)$	10110 (0.41)<br>11001 (0.29)<br>10101 (0.13)	22089
$^4T_1(2)$	01110 (0.36)<br>01101 (0.35)<br>10011 (0.13)	24088
$^4T_1(3)$	11010 (0.40)	24215

	10110 (0.23)<br>01110 (0.13)<br>01011 (0.09)	
$^2A_1$	11010 (0.24)<br>20001 (0.16)<br>20010 (0.13)<br>02010 (0.13)<br>00201 (0.13)<br>011010 (0.08)	26448
$^2A_2$	11010 (0.54)<br>20010 (0.24)<br>02010 (0.08)	28073
$^4T_1$(1)	01011 (0.62)<br>00111 (0.24)	34636
$^4T_1$(2)	10011 (0.79)<br>01101 (0.06)	35498
$^4T_1$(3)	00111 (0.61)<br>01011 (0.23)	39404

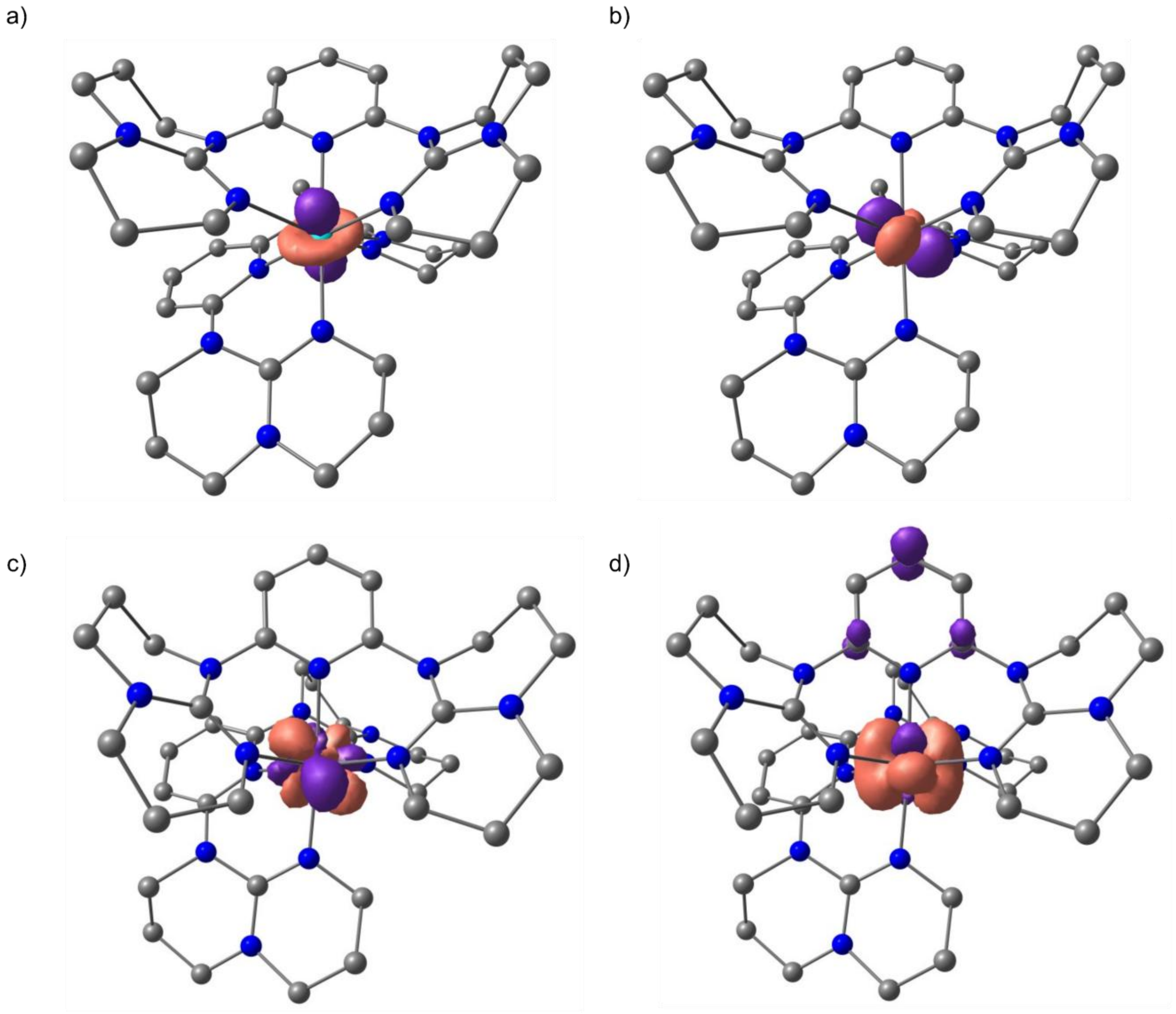

Figure S22. Frontier orbitals of (restricted) DFT-optimized **[V(dgpy)$_2$]$^{3+}$** a) HOMO (#200) and b) LUMO (#201) with isosurface values of 0.1. Spin density plots of c) lowest-energy singlet state of (unrestricted) DFT-optimized **[V(dgpy)$_2$]$^{3+}$** and d) the lowest-energy doublet state of DFT-optimized **[V(dgpy)$_2$]$^{2+}$**. α and β spin densities (orange/purple) are displayed with isosurface values of 0.008. Hydrogen atoms are omitted.

Table S10. Bond lengths [Å] and angles [deg] of **[V(dgpy)$_2$]$^{2+}$**, **[V(dgpy)$_2$]$^{3+}$** in their lowest-energy SF excited states obtained from DFT calculations.

	**DFT**		
	**[V(dgpy)$_2$]$^{2+}$**	**[V(dgpy)$_2$]$^{3+}$ (uks)**	**[V(dgpy)$_2$]$^{3+}$ (rks)**
V1-N1	2.129	2.071	2.053
V1-N2	2.072	2.126	2.168
V1-N3	2.146	2.083	2.064
V1-N4	2.162	2.101	2.064
V1-N5	2.163	2.136	2.170
V1-N6	2.133	2.068	2.051
N1-V1-N2	83.06	82.27	82.22
N1-V1-N3	93.76	93.56	94.67
N2-V1-N3	82.42	81.61	79.32
N5-V1-N4	81.45	82.38	79.16
N6-V1-N4	92.62	93.35	94.55
N6-V1-N5	80.22	81.60	82.25
N1-V1-N5	174.56	175.18	178.00
N2-V1-N6	175.42	175.50	178.00
N3-V1-N4	170.00	169.26	156.42
